# Abnormal gametogenesis induced by p53 deficiency promotes tumor progression and drug resistance

**DOI:** 10.1038/s41421-018-0054-x

**Published:** 2018-10-02

**Authors:** Chunfang Liu, Zhen Cai, Guoxiang Jin, Danni Peng, Bo-Syong Pan, Xian Zhang, Fei Han, Xiaohong Xu, Hui-Kuan Lin

**Affiliations:** 1 Department of Laboratory Medicine, Huashan Hospital, Shanghai Medical College, Fudan University, Shanghai, 200040 China; 2 Department of Cancer Biology, Wake Forest School of Medicine, Winston-Salem, NC 27157 USA; 3 Department of Molecular and Cellular Oncology, The University of Texas MD Anderson Cancer Center, Houston, TX 77030 USA; 4 Graduate Institute of Basic Medical Science, China Medical University, Taichung, 404 Taiwan; 5 Department of Biotechnology, Asia University, Taichung, 41354 Taiwan

## Abstract

The century-old embryonal/gametogenesis hypothesis of tumors could link diverse tumors’ malignant features together likely representing the real “stemness” of tumors. However, the genetic evidence to validate abnormal gametogenesis in tumors remains lacking. Here we show that p53 deficiency elicits abnormal gametogenesis from primordial germ cell-like stage to late oocyte-like stage and subsequent parthenogenetic activation. The similar upregulation of abnormal gametogenesis by p53 deficiency is observed both in p53^−/−^ mouse model and cultured cancer cells. Notably, germ cell-like cells isolated from distinct tumors from p53^−/−^ mice and cancer cell lines display potent tumorigenicity potential. Abnormal oogenesis induced by p53 deficiency and then spontaneous parthenogenetic activation endow tumors with imitated embryonic development, life cycle, and therapeutic resistance. Our study establishes the genetic evidence to support embryonal/gametogenesis theory of tumors and reveals a pivotal role of p53 in restricting abnormal gametogenesis that may represent a novel aspect for p53’s tumor suppression.

## Introduction

The century-old embryonal/gametogenesis theory of tumors proposed that tumors arise from germ cells and thus are in some way similar to the formation of gametes and fertilization^1–3^. Old extended the hypothesis postulating that the silenced gametogenic program is reactivated in tumors and serves as one of the driving forces for tumorigenesis^2,3^. It has been well known that the key malignant features of tumors are closely similar to those of embryonic/germ cells, such as immortalization, invasion, independence, a lack of adhesion, migration (corresponding to metastasis), demethylation, induction of meiosis (corresponding to aneuploidy), angiogenesis induction, and immune evasion^2,3^. The embryo/germ cell-like developmental axis could link a variety of tumors’ malignant features together that might represent the real “stemness” of tumors, thus making the hypothesis very attractive^2,3^. This hypothesis was partially supported by some experimental evidence^2–6^. First, earlier studies reported that embryonic antigens (such as human chorionic gonadotropin, HCG) and testis antigens known as cancer/testis antigens, such as Vasa and SCP3, are upregulated in human solid cancers and their upregulation is associated with disease progression and predicts poor prognosis^2–6^. Second, ectopic expression of germ cell genes in Drosophila drives brain tumor growth^7^. Third, our previous studies showed that germ cell-like cells may exist in cultured cancer cells^8,9^ and abnormal gametogenesis could be activated by chemical carcinogen^10^. However, no genetic mouse tumor models are utilized to identify the occurrence of germ cell traits in tumors and their potential roles in oncogenic processes. Moreover, if the concept is true, it is crucial to identify the signals that could drive the acquisition of germ cell traits during cancer development. Validating the critical concept and identifying the key signal regulating abnormal gametogenesis may gain marked advance in further understanding of cancer evolutions that may provide new avenues and paradigms for cancer targeting.

p53 is a central tumor suppressor that regulates diverse biologic processes, including cell cycle arrest, apoptosis, and senescence^11^. Interestingly, p53 not only plays a crucial role in maintaining genomic stability in somatic cells but also regulates meiosis and genomic stability in gametogenesis^12,13^. Therefore, we postulated that if activation of abnormal gametogenesis occurs during cancer development and is crucial for tumorigenesis, p53 may be a key regulator to restrict this process, thus contributing to its tumor suppression. To test this hypothesis, p53-deficient mice and cells were used to further validate the abnormal gametogenesis during cancer progression.

## Results

### p53 deficiency facilitates abnormal gametogenesis

**Fig. 1:**
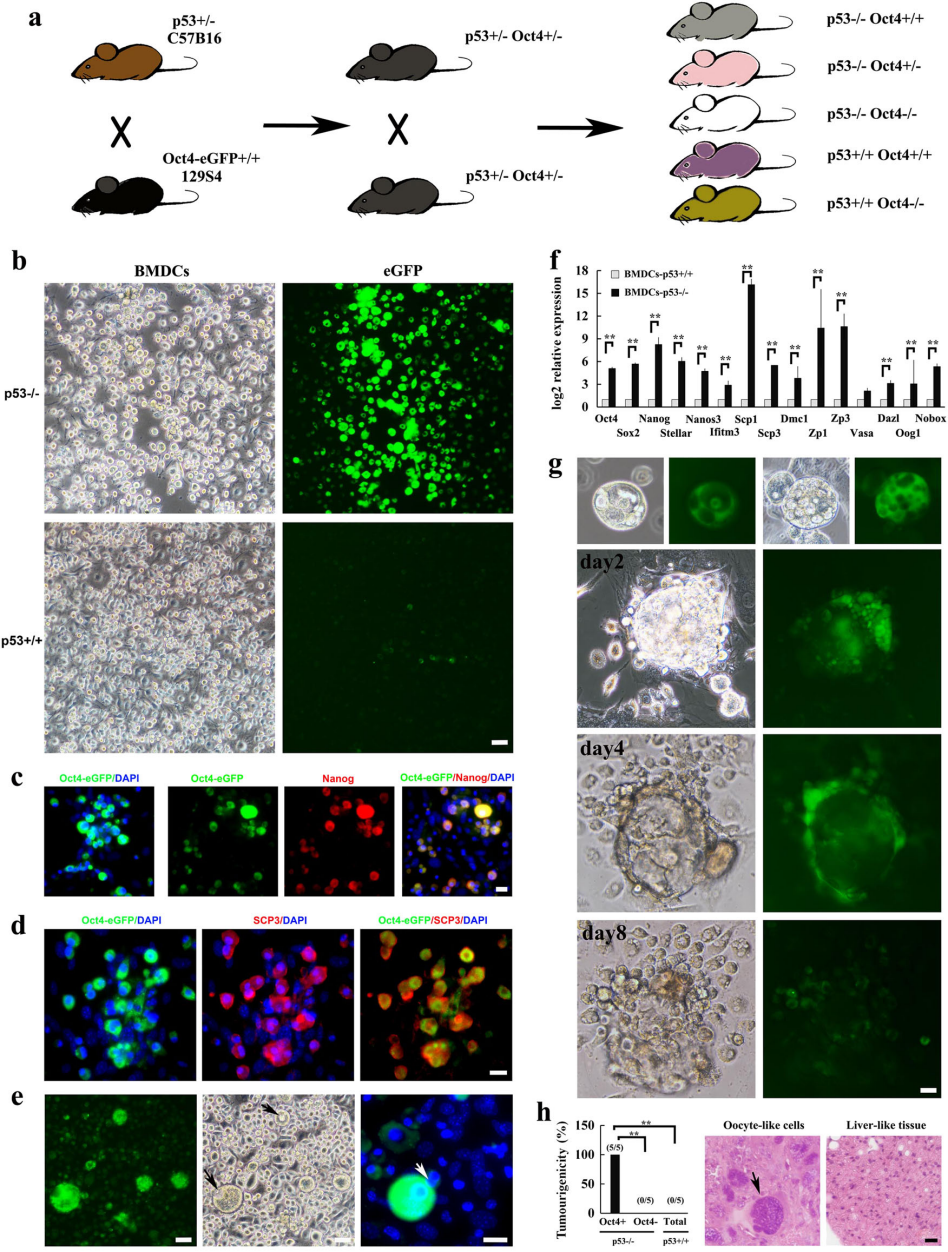
Abnormal gametogenesis is detected in p53^−/−^ BMDCs. **a** The mice-breeding scheme for gametogenesis study was shown. **b** Bright-field and Oct4-GFP fluorescence image of primary BMDCs derived from p53^−/−^ and p53^+/+^ mice were shown. **c** Oct4-GFP fluorescence, Nanog staining image, and DAPI staining image showed the morphology and germ cell marker expression of germ cell-like cells in p53^−/−^ BMDCs. **d** Oct4-GFP fluorescence and meiosis marker SCP3 staining image in p53^−/−^ BMDCs. **e** Bright-field, Oct4-GFP fluorescence image and DAPI staining of p53^−/−^ BMDCs showed the oocyte-like cells with germinal vesicle (GV)-like structures (black arrow) and oocyte-like cells with a polar body (PB)-like structure (white arrow). **f** The qRT-PCR for a series of germ cell markers, including early germ cell markers (Oct4, Sox2, Nanog, Stellar, Nanos3, and Ifitm3), meiosis markers (Scp1, Scp3, and Dmc1), and late germ cell markers (Vasa, Dazl, Oog1, and Nobox) in paired primary BMDCs derived from p53^−/−^ and p53^+/+^ mice (*n* = 3). **p < 0.01. **g** Bright-field and Oct4-GFP fluorescence image showed the embryo-like structures at the different developmental stages in p53^−/−^ BMDCs were seen after sub-culturing at different culture times. **h** Oct4-GFP^+^ (100 cells) and Oct4-GFP^−^ (100 cells) cells isolated from p53^−/−^ BMDCs or p53^+/+^ BMDCs (~500 000 cells) with six passage culture were injected subcutaneously into nude mice (5 mice/each group) for tumorigenicity assay (*n* = 5). **p < 0.01. The representative tumor sections from Oct4-GFP^+^ p53^−/−^ BMDCs stained with hematoxylin and eosin (H&E). The oocyte-like tumor cell was indicated with arrow (scale bar = 20 μm)

To examine the acquisition of germ cell-like cells upon p53 deletion during cancer development, we crossed p53^+/−^ mice with Oct4-GFP knock-in reporter mice, which harbor IRES-GFP fusion cassette downstream of the stop codon of the *Oct4* (*Pou5f1*) gene, and the resulting mice were further intercrossed to generate p53^+/+^/Oct4-GFP^+/+^ and p53^−/−^/Oct4-GFP^+/+^ mice for further study (Fig. 1a). Since bone marrow-derived cells (BMDCs) were reported to have potential to generate germ cells under certain conditions^14^, we first isolated and cultured BMDCs from these mice and monitored Oct4 expression through green fluorescent protein (GFP) fluorescence signal as a surrogate for germ cell-like cells, as *Oct4* is a key gene involved in the specification and development of germ cells^15,16^. Compared with the primary p53^+/+^ BMDCs, Oct4-GFP^+^ germ cell-like cells were markedly increased in the primary p53^−/−^ BMDCs (~50–250 cell clusters per bone marrow) after 4 weeks of culture (Fig. 1b, Supplementary Fig. S1a, Supplementary Table S1). The similar phenomenon was observed in BMDCs from three independent p53^−^^/−^ mice (Supplementary Table S1). A series of germ cell-like cells from primordial germ cells (PGCs) to later oocyte-like cells^17^ could be observed and colocalized with Oct4-GFP signal in cultured p53^−/−^ BMDCs (Fig. 1b–e), as determined by the expression of a series of germ cell special markers^15,16^, including Oct4, nonspecific alkaline phosphatase (AP), Nanog, Nanos3, Sox2, deleted in azoospermia-like (DAZL), and growth differentiation factor (GDF9) in these cells (Fig. 1c, Supplementary Fig. S1b-d).

The observation of a series of germ cell-like developmental events^17^, such as proliferation, forming cell clusters (Supplementary Fig. S1e), departing from dish (similar to PGC migration) (Supplementary Fig. S1e), and even meiosis (Fig. 1d, e), in germ cell-like cells of p53^−/−^ BMDCs further endorsed their similarity to natural germ cells. The meiotic events found in germ cell-like cells included the expression of synaptonemal complex protein 3 (SCP3), an excellent marker for the meiotic entry (Fig. 1d), germinal vesicle (GV)-like structures (Fig. 1e, left panel) and polar body (PB)-like structures (Fig. 1e, right panel)^15^. It is possible the oocyte-like cells could undergo meiosis I, although there is no evidence for the completion of meiosis II. Quantitative RT-PCR (qRT-PCR) results showed that the expression of various germ cell-specific markers from early to late developmental stages abruptly increased in p53^−/−^ BMDCs compared with p53^+/+^ BMDCs (Fig. 1f), further confirming that p53 deletion promotes the formation of germ cell-like cells.

The most important function of oocyte is to generate embryo via fertilization or parthenogenetic activation^15^. The latter was reported in ovary cancer or in vitro-cultured oocytes^15,17,18^. Notably, parthenogenetic embryo-like structures representing different developmental stages could be frequently observed in p53^−/−^ BMDCs (Fig. 1g, Supplementary Fig. S1c) and could further give rise to new offspring cells (Fig. 1g, Supplementary Movie S1), but was rarely detected in p53^+/+^ BMDCs.

There is an intimate relationship between germ cells and tumorigenesis^3^. Early germ cells before embryonic day (E) 12.5 and oocytes undergoing parthenogenesis are susceptible to the formation of teratomas^3,19,20^. In clinical cases, the study in testicular tumors, ovary tumors, and germ cell tumors out of genital ridge also supports the notion that germ cells may contribute to tumorigenesis, including teratomas, germ cell tumors, and embryonal carcinoma^3^. We then sorted Oct4-GFP^+^ and Oct4-GFP^−^ p53^−/−^ BMDCs under six passages of the culture and subcutaneously injected them to nude mice for tumor engraftment assay. The 100 Oct4-GFP^+^ p53^−/−^ BMDCs could readily formed tumor in all nude mice tested within 4 months (Fig. 1h). By contrast, 100 Oct4-GFP^−^ p53^−/−^ BMDCs and 5 × 10^5^ unsorted p53^+/+^ BMDCs failed to engraft in nude mice (Fig. 1h). The tumors derived from Oct4-GFP^+^ p53^−/−^ BMDC cells were sarcoma-like with few PGC-like tumor cells detected and often accompanied by hepatic-like tissues (3/5 mice) and large tumor cells (2/5 mice) (Fig. 1h, Supplementary Fig. S1f, g), which were positive for Oct4 and DAZL (Supplementary Fig. S1g) resembling oocyte or early parthenogenetic preimplantation embryo in morphology and marker expression. The other two p53^−/−^ BMDCs also showed the ability to form similar sarcoma-like tumors (Supplementary Fig. S1h, Supplementary Table S1). Thus, Oct4-GFP^+^ p53^−/−^ BMDCs have tumorigenicity potential. Together, these findings suggest that p53 serves as a barrier to restrict abnormal gametogenesis in somatic tissue-derived cells.

### Germ cell-like cells exist in p53^−/−^ mice

**Fig. 2:**
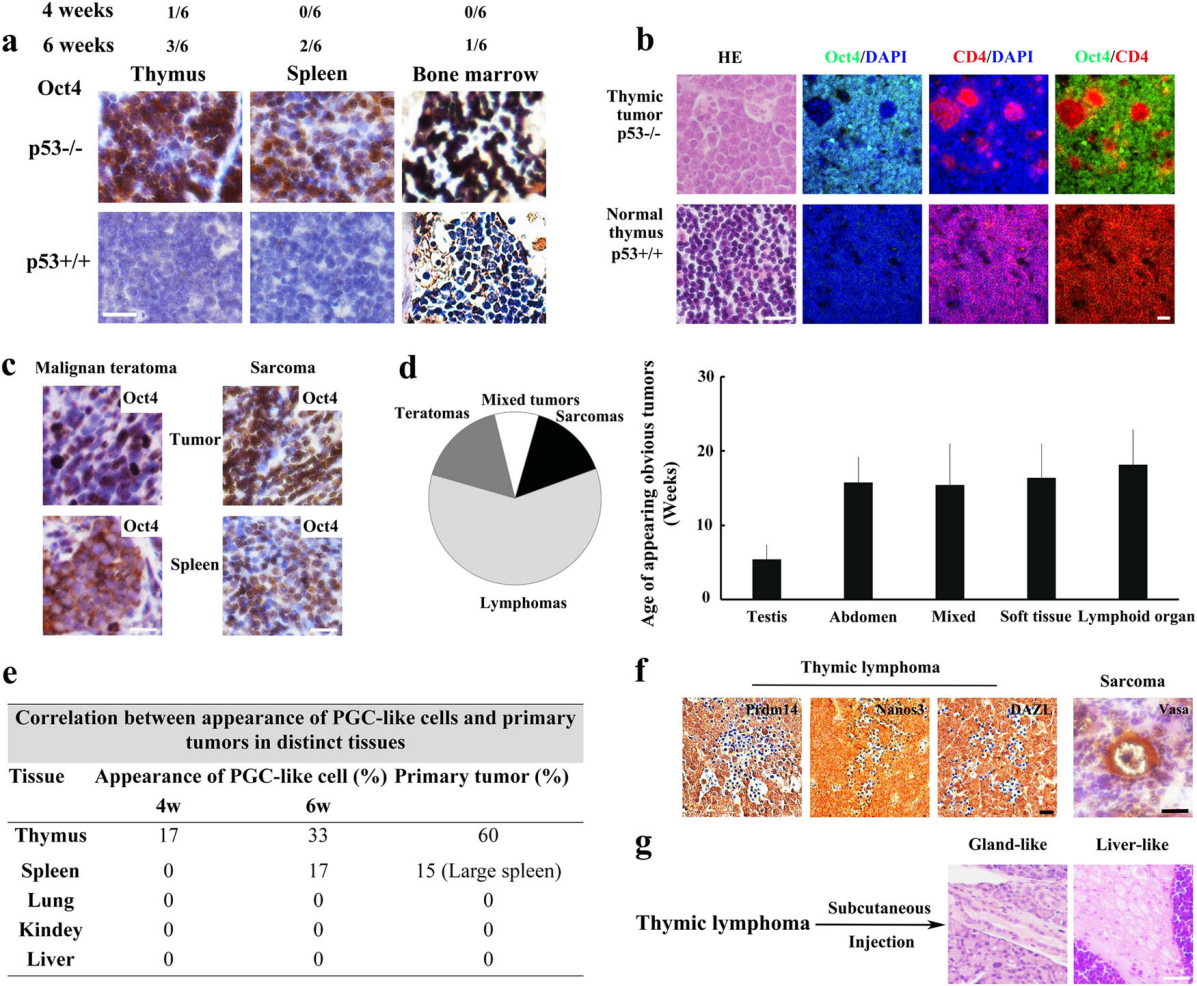
Extensive upregulation of abnormal gametogenesis is found in tumors from p53^−/−^ mice. **a** Sections of paired normal tissues derived from p53^−/−^ and p53^+/+^ mice were stained with Oct4 antibody. The relative ratio of obvious Oct4^+^ cells appeared in the p53^−/−^ mice with different ages was shown. **b** Sections of paired thymic lymphoma of p53^−/−^ mouse and normal thymus of p53^+/+^ mouse were stained with H&E or antibodies against the indicated proteins. **c** Sections of malignant teratoma, spleen, and sarcoma from p53^−/−^ mice were stained with Oct4 antibody. **d** The pie chart showed the spontaneous tumor types with different percentage in p53^−/−^ mice. The age of tumor formation was counted at the appearance of obvious tumors. The plot showed the age of appearing obvious tumors in the different spontaneous tumor types in p53^−/−^ mice. **e** The correlation between the appearance of PGC-like cells and tumor formation in a variety of tissues from p53^−/−^ mice. **f** Section from spontaneous tumors of p53^−/−^ mice were stained with antibody against Prdm14, Nanos3, Vasa, or H&E. Section of sarcoma stained with Vasa antibody showed an oocyte-like tumor cell (arrow). **g** Sections were stained with H&E (scale bar = 20 μm)

It is well established that p53^−/−^ mice display spontaneous tumors with multiple tumor types within 9 months^21^. We therefore used the p53^−/−^ mouse model to further investigate whether abnormal gametogenesis occurs in vivo tumors and contributes to tumorigenesis. As early as 4 weeks, Oct4^+^ PGC-like cells were observed to enrich in a few thymi of p53^−/−^ mice. Compared to p53^+/+^ mice (six paired mice), the Oct4^+^ PGC-like cells were highly enriched in thymus, bone marrow, and spleen (Fig. 2a), but not in kidney, liver, muscle, and lung (Supplementary Fig. S2a), from p53^−/−^ mice before obvious tumors developed, suggesting that the enrichment of PGC-like cells in p53^−/−^ mice was age- and tissue-sensitive. Notably, Oct4^+^ PGC-like cells abruptly increased in all p53^−/−^ mice with spontaneous tumors especially in lymphomas, malignant teratomas, and enlarged spleen (Fig. 2b, c, Supplementary Fig. S2b, c), but not in those paired normal tissues from p53^+/+^ mice except for testis.

Consistent with the earlier reports^21,22^, tumors derived from p53^−/−^ mice were mostly lymphomas (thymus and lymph node) (Fig. 2d, Supplementary Table S2), followed by malignant teratomas (testis and abdomen) (Supplementary Fig. S2d) and sarcomas (subcutis) (Supplementary Fig. S2e), and some of them were accompanied with enlarged spleen (Fig. 2b, Supplementary Table S2). The average age was about 5 weeks for the appearance of obvious tumors in testis, 15 weeks in abdomen, 16 weeks in mixed tissue, 16 weeks in soft tissue, and 18 weeks in thymus (Fig. 2d, Supplementary Table S2). All p53^−/−^ mice died within 9 months, but no tumor was observed in p53^+/+^ mice at this time. The spontaneous tumors in p53^−/−^ mice were also age- and tissue-sensitive. In essence, the tissues with PGC-like cell enrichment readily gave rise to spontaneous tumors (Fig. 2e). PGCs often were considered as cell origin of the teratomas^1–3^. Our above finding that germ cell-like cells of BMDCs could give rise to sarcoma suggests that the spontaneous sarcoma might link to the appearance of PGC-like cells.

In the spontaneous lymphomas, two main distinct subpopulations of tumor cells were observed, namely, CD4^+^ lymphocyte-origin tumor cells and Oct4^+^ germ cell-like cells (Fig. 2b). The ratio of germ cell-like cells varied from about 20 to 90% in thymic lymphomas but was almost undetectable in normal thymus (Fig. 2b, Supplementary Fig. S2b, 2c). The PGC-like cells were further validated by the expression of other germ cell-specific markers, such as Prdm14, Nanos3, and DAZL (Fig. 2f). The spontaneous sarcomas resembled markedly the xenograft tumors derived from Oct4-GFP^+^ p53^−/−^ BMDCs in histological images (Supplementary Fig. S2e), which contained a low level of Oct4^+^ PGC-like cells. Oocyte-like cells were observed frequently in sarcomas but not in lymphoma and teratomas, as determined by oocyte-specific marker Vasa expression and morphology (Fig. 2f). Mature tissues, such as hepatic-like pattern and gland-like pattern could be observed in some of sarcomas from p53^−/−^ mice as well as xenograft tumors derived from the thymic lymphomas (Fig. 2g, Supplementary Table S2), resembling early PGCs in multipotency^19^. Together, the findings suggest that p53 deficiency drives abnormal gametogenesis that may be involved in spontaneous tumor development in p53^−/−^ mice.

### Germ cell-like cells from p53^−/−^ mice display tumorigenicity potential

**Fig. 3:**
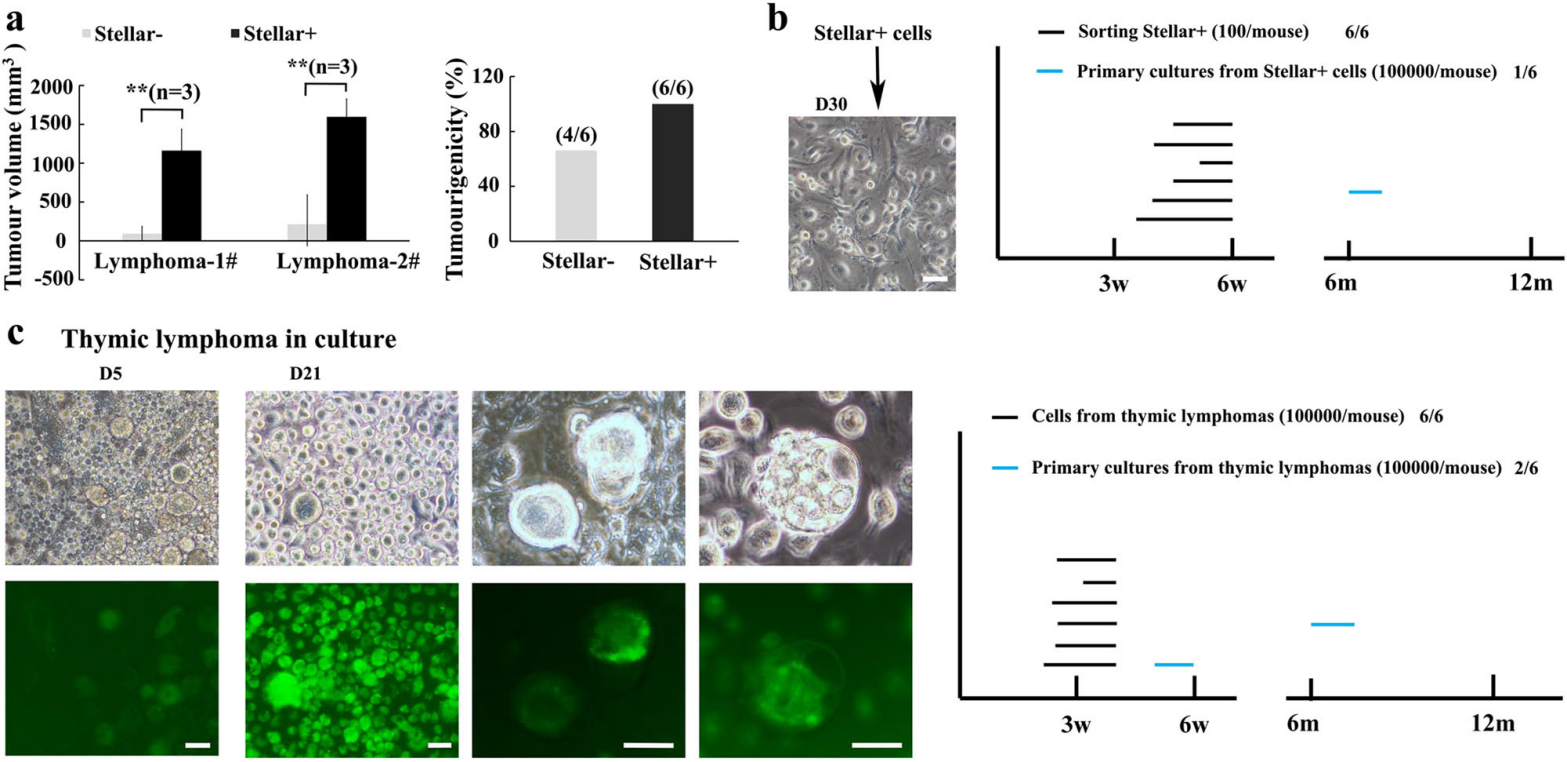
PGC-like cells from p53^−/−^ mice show strong tumorigenicity potential. **a** In all, 100 Stellar^−^ or Stellar^+^ cells sorted from two different p53^−/−^ lymphomas by FACS were injected subcutaneously into nude mice, and tumor volume was measured at 6 weeks after injection. The plots showed relative tumor volume from paired sorted Stellar^−^ and Stellar^+^ cells within 6 weeks (*n* = 3). **p < 0.01. **b** 100 Stellar^+^ cells after cultured in dish for 30 days. The cultures from the Stellar^+^ cells after cultured for 30 days were injected subcutaneously into nude mice and tumorigenicity was analyzed. The plot showed the distinct tumorigenicity of sorting Stellar^+^ cells and cultured Stellar^+^ cells. **c** Bright-field and Oct4-GFP fluorescence image of primary cultured thymic lymphomas at different time points. The plot showed the distinct tumorigenicity of total thymic lymphoma cells and primary cultures from thymic lymphoma after cultured for 21 days (scale bar = 40 μm in **b** and 20 μm in **c**)

The spontaneous thymic lymphomas in p53^−/−^ mice were used as a model to study the traits of germ cell-like cells. To further validate the tumorigenicity of the germ cell-like cells, we then isolated Stellar^+^ cells representing germ cell-like cells from the thymic lymphomas, as Stellar is a germ cell-specific marker^15^, and Stellar^−^ cells as somatic tumor cells, and subcutaneously injected these cell populations to nude mice (100 cells/mouse) for tumorigenicity assay. Stellar^+^ cells gave rise to large tumor in all mice 6 weeks after injection, but Stellar^−^ subpopulation failed to do so or only gave rise to very small tumor (Fig. 3a), indicating that germ cell-like cells, but non-germ cell-like cells, have strong tumorigenicity potential in vivo. Liver-like tissues and fat-like tissues were also observed in some of these tumors derived from sorted Stellar^+^ cells (Supplementary Fig. S2f). Our data indicate that germ cell-like cells play a crucial role in tumorigenicity.

To gain better understanding of PGC-like cells, 100 Stellar^+^ cells isolated from thymic lymphomas in p53^−/−^ mice were then cultured in vitro. Upon cultured in regular culture medium, the Stellar^+^ cells attached to the dish and quickly differentiated into fibroblast-like somatic cells with few germ cell-like cells (Fig. 3b). Remarkably, the tumorigenicity of Stellar^+^ cell-derived primary culture abruptly declined (Fig. 3b). The thymic lymphomas cells from p53^−/−^/Oct4-GFP^+/+^ mice were also isolated for primary cell culture. The Oct4-GFP^+^ cells attached to the dish and further grew, whereas Oct4-GFP^−^ cells failed to attach to the dish and died within 2 weeks of culture (Fig. 3c). Three weeks after the culture, the Oct4-GFP^+^ cells derived from the thymic lymphomas could also differentiate into Oct4-GFP^−^ fibroblast-like cells likely representing somatic tumor cells and gave rise to germ cells at the later developmental stages and their early embryo-like derivatives in the primary culture (Fig. 3c). Similar to primary culture of Stellar^+^ cells, the primary culture of lymphoma cells also led to the abrupt decrease in tumorigenicity (Fig. 3c), likely resulting from the loss of early PGC-like cells during the culture. Collectively, these findings indicate that the PGC-like cells could differentiate into both later germ cells and somatic cells and play an important role in tumorigenicity.

### p53 restricts the formation of early germ cell-like cells

**Fig. 4:**
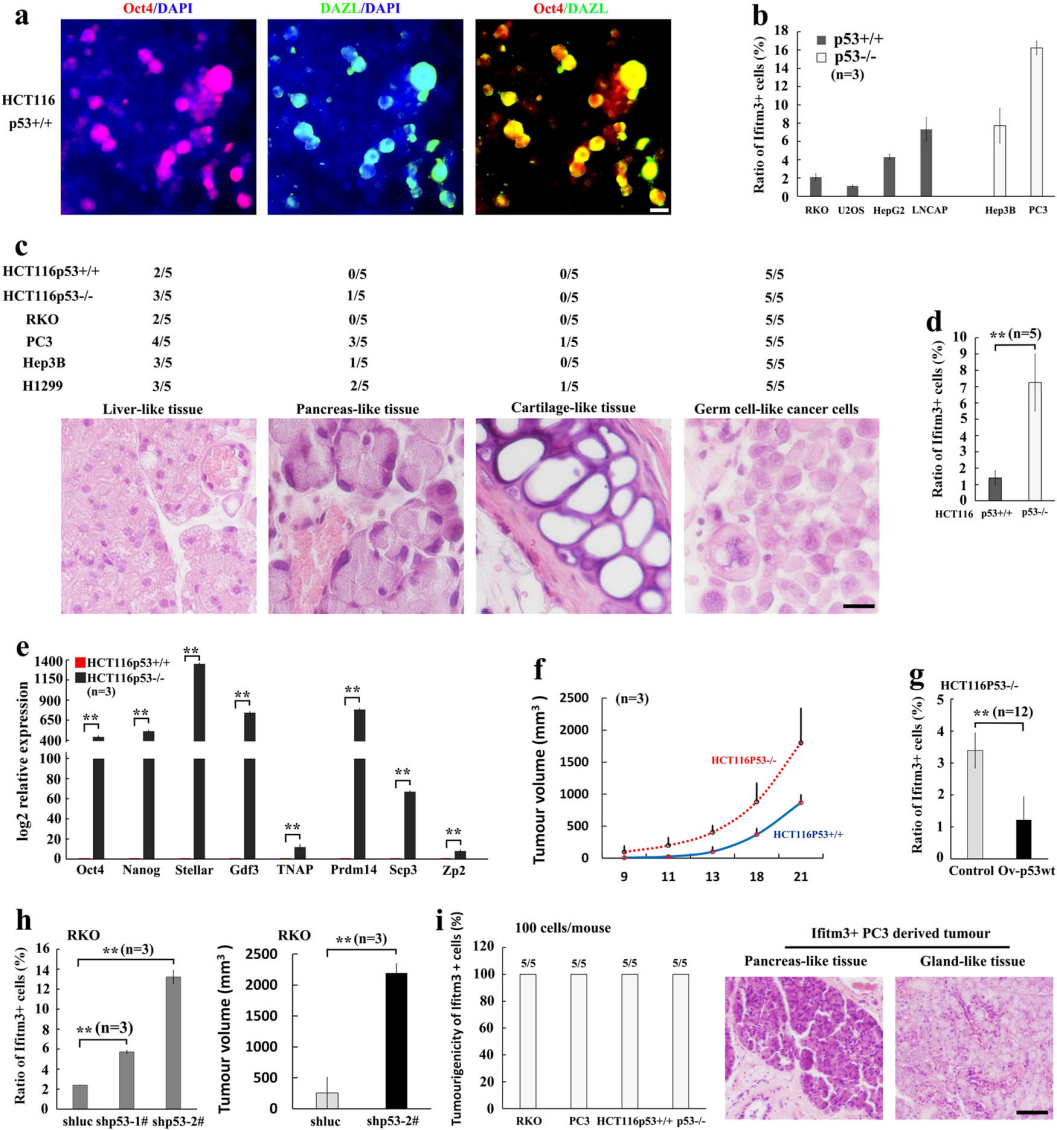
Germ cell-like cells are enriched in diverse human cancer cell lines. **a** Immunofluorescence assay showed that expression and colocalization of Oct4 and DAZL in a subset of cultured p53^+/+^ HCT116 cells. **b** The percentages of Ifitm3^+^ cells in diverse cancer cell lines with or without p53 were analyzed by flow cytometry (*n* = 3). **c** Diverse cancer cell lines were injected subcutaneously into nude mice (1 × 10^5^ cells/mouse), and tumors were collected for H&E staining. The incidence for liver-like tissues, pancreas-like tissues, and cartilage-like tissues was counted. Representative tumor sections from PC3 cells stained with H&E were shown. **d** The ratio of ifitm3^+^ in the isogenic wild-type and p53^−/−^ HCT116 cancer cell lines was analyzed by flow cytometry (*n* = 5), **p < 0.01. **e** The relative expression of a series of germ cell-related markers in paired p53^+/+^ and p53^−/−^ HCT116 cells was analyzed by RT-PCR. **f** The plot showed the tumor growth curve from nude mice injected with p53^−/−^ HCT116 and p53^+/+^ HCT116 cells. **g** The relative ratio of Ifitm3^+^ in p53^−/−^ HCT116 cells with or without p53 restoration from multiple single-cell clones was analyzed by flow cytometry (*n* = 12). **p < 0.01. **h** The relative ratio of Ifitm3^+^ cells in RKO cells with control or p53 knockdown was analyzed by flow cytometry (*n* = 3). **p < 0.01. Tumor volume from nude mice injected subcutaneously with RKO cells with control or p53 knockdown was shown (*n* = 3). **p < 0.01. **i** A total of 100 Ifitm3^+^ cells isolated from diverse cancer cell lines were injected subcutaneously into nude mice for tumorigenicity assay (*n* = 5). The tumor sections from PC3 cells were stained with H&E (scale bar = 20 μm)

The findings that overexpression of germ cell markers, such as, Oct4, SSEA1 (a germ cell marker in human), Nanog, Ifitm3, and c-Kit has been documented in numerous human cancers and some of these genes are shown to play important roles in tumorigenesis^23–26^ raise the possibility that the appearance of germ cell-like cells is common in human cancers. We thus sought to determine whether germ cell-like cells could be detected in diverse human cancer cell lines with distinct genetic backgrounds. Earlier germ cell-like cells, as determined by co-expression of Oct4 and DAZL as well as expression of Ifitm3, could be detected both in wild-type (RKO, U2OS, LNCaP, and HCT116) and p53-null (Hep3B, PC3, and p53^−/−^ HCT116) cancer cell lines (Fig. 4a, b). Notably, xenograft tumors derived from various cancer cell lines subcutaneously injected to nude mice showed the existence of germ cell-like cells in conjunction with mature tissues (Fig. 4c), suggesting the existence of cells with functions resembling germ cells. The findings suggest that germ cell-like cell formation is common in cancer cell lines and p53 may not be the only player to regulate abnormal gametogenesis.

To further validate the role of p53 in abnormal gametogenesis, we used the isogenic wild-type and p53^−/−^ HCT116 colon cancer cell lines. The analysis of flow cytometry and quantitative reverse transcription-PCR in p53^−/−^ HCT116 cells and p53^+/+^ HCT116 cells revealed that p53 deletion also upregulated the ratio of germ cell-like cancer cells in cultured cancer cells (Fig. 4d, e). The tumorigenicity potential was higher in p53^−/−^ HCT116 cells compared with p53^+/+^ HCT116 cells (Fig. 4f). Restoration of p53 expression in p53^−/−^ HCT116 cells reduced the percentage of Ifitm3^+^ germ cell-like cells (Fig. 4g). By contrast, knockdown of p53 in RKO cells enriched earlier Ifitm3^+^ germ cell-like cell populations and enhanced tumorigenicity (Fig. 4h). After subcutaneous injection, 100 Ifitm3^+^ germ cell-like cells sorted from various cancer cell lines could all give rise to tumors, and some of which exhibited high level of mature tissues, especially in those from the PC3 cancer cell line (Fig. 4i). Thus, p53 deficiency promotes abnormal gametogenesis with tumorigenicity potential in diverse cancer cell lines, accompanied by the increase in tumorigenicity in vivo.

### p53 deficiency promotes abnormal oogenesis in cancer

**Fig. 5:**
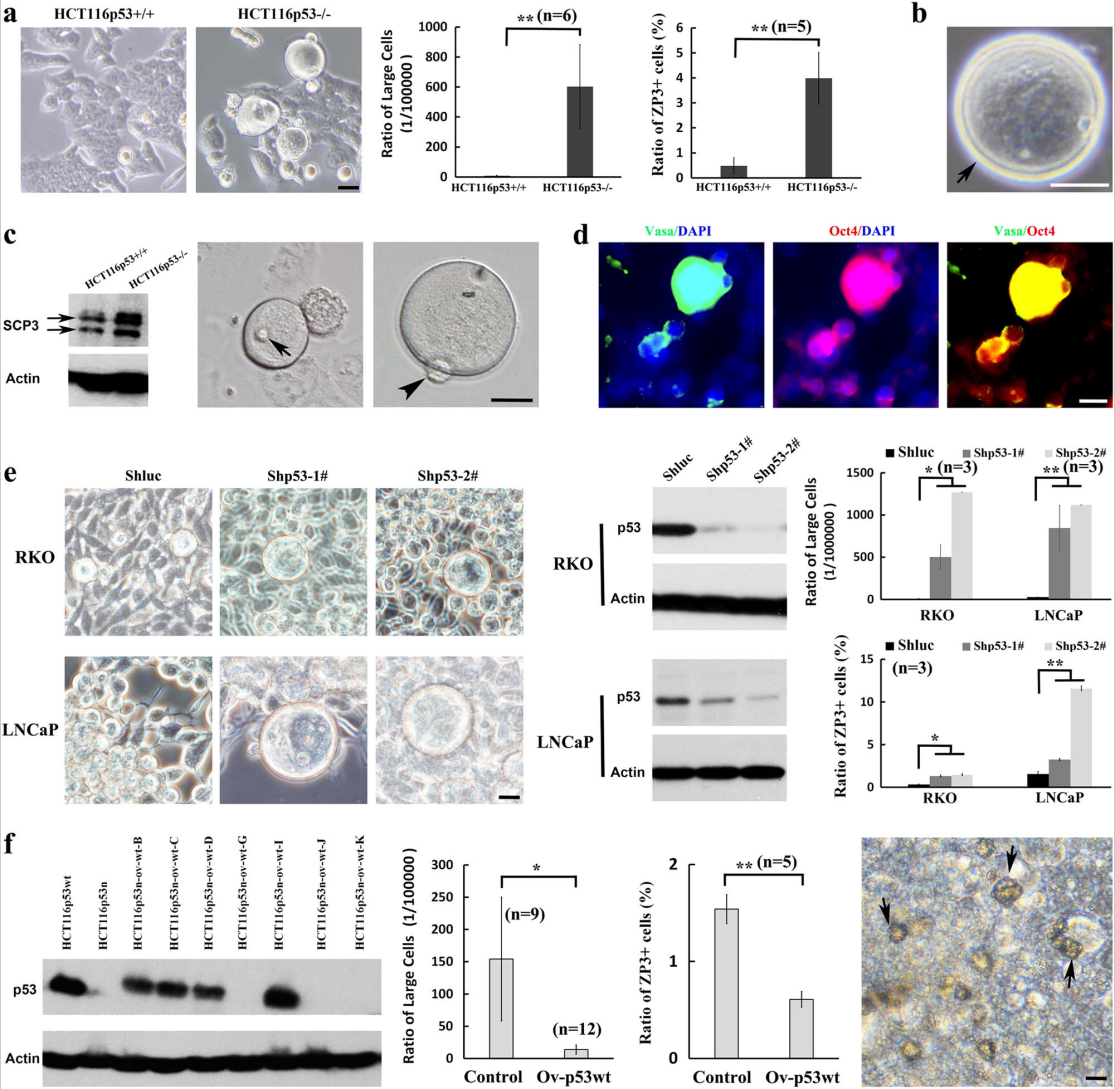
p53 deficiency dictates oocyte-like large cell formation. **a** Representative bright-field images of paired p53^+/+^ and p53^−/−^ HCT116 cultures were shown. The oocyte-like large cells were frequently observed in p53^−/−^ HCT116 cultures (especially >25 μm in diameter). The oocyte-like tumor cells larger than 25 μm in diameter were counted under high-power microscope in p53^+/+^ and p53^−/−^ HCT116 cultures (*n* = 6). The relative ratio of ZP3^+^ cells in p53^+/+^ and p53^−/−^ HCT116 cultures were analyzed by flow cytometry (*n* = 5). **p < 0.01. **b** Representative bright-field image of an oocyte-like cell with zona pellucida-like membrane (arrow) in p53^−/−^ HCT116 cultures was shown. **c** The immunoblotting showed the expression of meiosis entry related protein SCP3 in p53^+/+^ and p53^−/−^ HCT116 cells. The oocyte-like tumor cells with GV-like structure (arrow) or polar body-like structure (arrow head) was detected in p53^−/−^ HCT116 cultures. **d** Immunostaining showed the expression of Oct4 and Vasa in oocyte-like large cells of p53^−/−^ HCT116 cultures. **e** Bright-field images showed oocyte-like cell appeared in RKO or LNCaP cells with p53 knockdown. The p53 immunoblotting was shown in RKO or LNCaP with control and p53 knockdown. The relative ratio of oocyte-like large cells or ZP3^+^ cells in RKO or LNCaP with control and p53 knockdown was analyzed by flow cytometry (*n* = 3). *p < 0.05, **p < 0.01. **f** Multiple single-cell clones were selected from p53^−/−^ HCT116 cells transfected with p53 for immunoblotting assay. The relative ratio of oocyte-like large cells (*n* = 9 and 12) and ZP3^+^ cells (*n* = 5) in p53^−/−^ HCT116 cells with or without p53 restoration from multiple single-cell clones were analyzed. *p < 0.05, **p < 0.01. Bright-field image showed the appearance of apoptotic cells in the p53^−/−^ HCT116 with p53 restoration (scale bar = 20 μm)

During oogenesis, PGCs migrate to gonad and differentiate into primary oocytes (primitive female germ cells, ~25 μm in diameter), which further differentiate to mature oocyte following meiosis^15^. Our findings that the appearance of oocyte-like large cells in cultured tumor cells and tumor tissues are reminiscent of the large cancer cells detected in human clinical cancers, which are frequently associated with p53 functional defects, higher pathological grades, and poor prognosis ^5,27^. Therefore, we asked the question whether p53 may also restrict abnormal gametogenesis at oogenesis developmental point in cancer cells. Notably, the oocyte-like large cells (>25 μm in diameter), which may represent oocyte cells at more mature stage, markedly increased in cultured p53^−/−^ HCT116 cells compared with p53^+/+^ HCT116 cells (Fig. 5a). The percentage of oocyte-like large cells was about 0.8% in p53^−/−^ HCT116 and 0.008% in p53^+/+^ HCT116 cells (Fig. 5a). The ratio of ZP3^+^ cells, an oocyte-specific marker, was about 1.5% in p53^−/−^ HCT116 cells, but was barely detected in p53^+/+^ HCT116 cells (Fig. 5a). Importantly, the ratio of oocyte-like large cells and ZP3^+^ cell populations were often higher in p53-null cancer cell lines (hep3B, PC3, and H1299 cells) than those from wild-type p53 cells (HepG2 and RKO) (Fig. 5a, Supplementary Fig. S3a).

Most of the oocyte-like large cells could reach 40–55 μm in diameter, and some of these large cells possessed a zona pellucida-like membrane (Fig. 5b). The oocyte-like large cells were also supported by the appearance of the germ cell-like cells at the developmental stages from early germ cells to oocytes (Fig. 5 c, d, Supplementary Fig. S3b-e), even at the meiotic stages, such as oocyte-like cells with GV-like structure or a PB-like structure (Fig. 5c) in cultured p53^−/−^ HCT116 cells^15^, and the expression of specific oocyte marker genes (Supplementary Fig. S3d), suggesting that the oocyte-like cells could go through meiosis I, although there is no evidence for the completion of meiosis II. While the expression of SCP3, an excellent marker for the meiotic entry, could be detected both in p53^+/+^ and p53^−/−^ HCT116 cells, its expression was much higher in p53^−/−^ HCT116 cells (Fig. 5c, Supplementary Fig. S3c). Interestingly, the oocyte-like cells before the GV stage and at early GV stage could be observed in cultured p53^+/+^ HCT116 cells, indicating that the oocyte-like cells might be eliminated during the further development in p53^+/+^ HCT116 cultures.

To further validate the role of *p53* gene in restricting the formation of oocyte-like large cells, we knocked down p53 in RKO and LNCaP cancer cell lines with functional p53 expression. The formation of oocyte-like large cells markedly increased upon p53 knockdown (Fig. 5e). Of note, restoration of p53 in p53^−/−^ HCT116 cells attenuated the formation of oocyte-like large cells (Fig. 5f, Supplementary Fig. S3f), accompanied by apoptosis induction (Fig. 5f), indicative of the vital role of p53 in eliminating abnormal oocyte-like cells in cancer cells. The findings are reminiscent of previous studies that p53 family proteins could prevent abnormal oocytes from surviving under radiation-induced meiotic checkpoint during oocyte development^12,13,28^. Our findings suggest that p53 serves as a barrier to restrict abnormal oogenesis in human cancer cell lines likely acting through the meiotic checkpoint.

### Abnormal oogenesis induced by p53 loss contributes to life cycle and therapeutic resistance

**Fig. 6:**
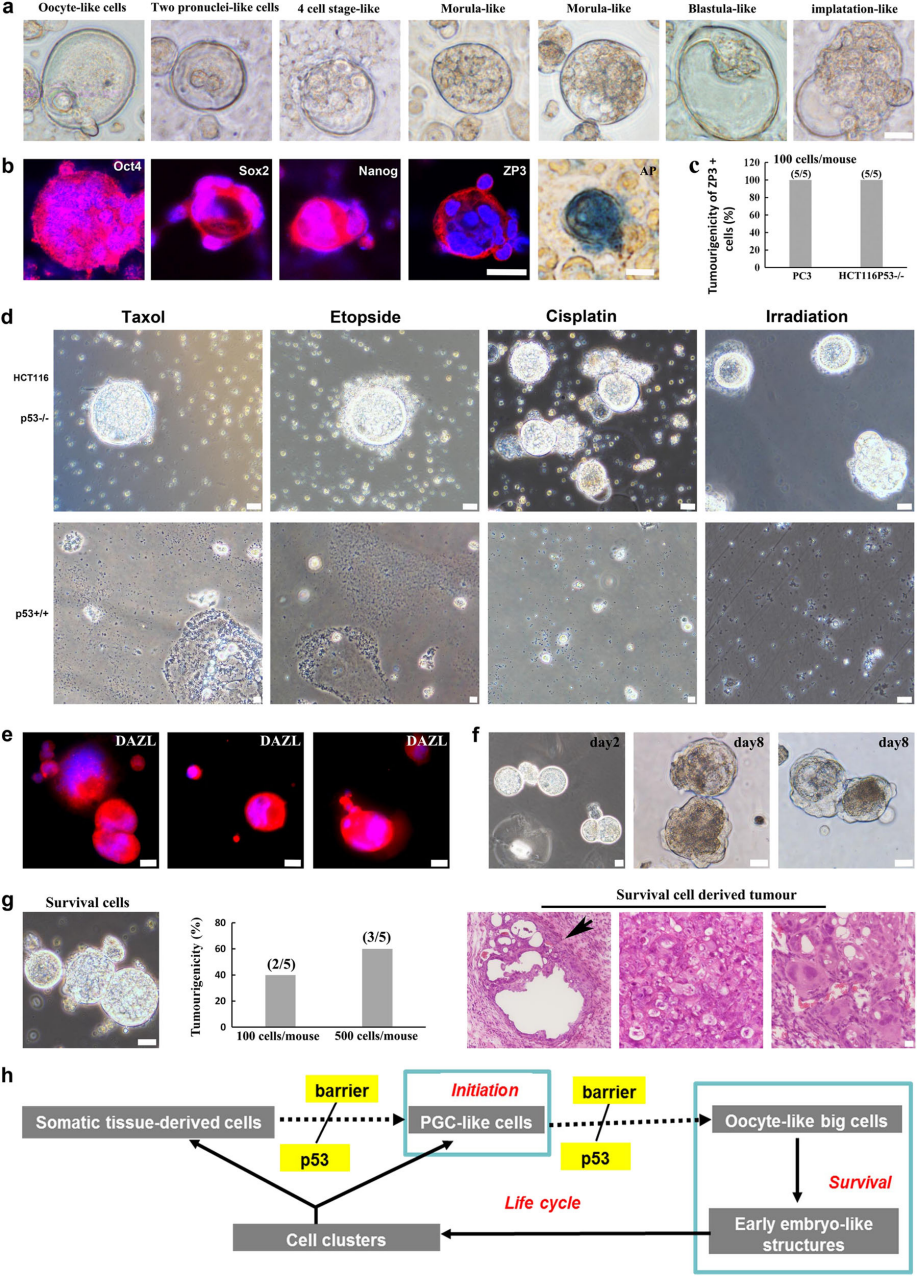
Abnormal oogenesis induced by p53 loss contributes to life cycle and therapeutic resistance. **a** Bright-field image of an oocyte-like cell and a series of embryo-like structures at different developmental stages in p53^−/−^ HCT116 cultures were shown. Somatic cancer cells were observed to derive from the embryo-like structures. **b** Cultured p53^−/−^ HCT116 cancer cells with embryo-like structures were stained with antibody against indicated proteins or AP. **c** Tumor incidence from nude mice injected with 100 ZP3^+^ cells isolated from PC3 and p53^−/−^ HCT116 cultures was shown. **d** Bright-field images showed the surviving cells from paired p53^+/+^ and p53^−/−^ HCT116 cells 4 weeks after distinct genotoxic treatment or 16 days after γ-irradiation. **e** The surviving cells after taxol treatment were stained with antibody against DAZL (a specific marker for germ cell and early preimplantation embryo). **f** Surviving p53^−/−^ HCT116 cells after treatment with taxol were cultured in semisolid medium, and bright-field images of spheres at day 2 and day 8 after culturing were shown. The new offspring cancer cells were observed to derive from surviving large cells at day 8 cultured in semisolid medium. **g** The surviving cells in p53^−/−^ HCT116 cells 4 weeks after taxol treatment were shown in the bright field. The surviving large cells were injected subcutaneously into nude mice for tumorigenesis assay (*n* = 5). The tumor incidence within 4 months was shown. Tumor sections stained with H&E showed the embryonic body-like structures (arrow) and germ cell-like cells. **h** The working model showed p53 deficiency promotes abnormal life cycle and gametogenesis (scale bar = 20 μm)

As oocytes contribute to reproduction^15^, it will be of interest to assess the functional relevance of the acquisition of oocyte-like cells during tumor progression. As expected, the early parthenogenetic embryo-like structures at different developmental stages, including 2- to 16-cell stage, morula, and blastocyte, could readily be observed in cultured p53^−/−^ HCT116 cells (Fig. 6a), but hardly detected in p53^+/+^ HCT116 cells. The early preimplantation embryo-like structures were positive for Oct4, Sox2, Nanog, ZP3, and AP staining (Fig. 6b), consistent with cultured early embryo^12,13,28^. The majority of these preimplantation embryo-like structures was degenerated or lost upon further subculture in regular culture medium, whereas about 20% of them in regular culture medium (Fig. 6a, Supplementary Fig. S4a, Supplementary Movie S2) and about 70% of them in semisolid culture medium (Supplementary Fig. S4a, Supplementary Movie S3) could give rise to new offspring cancer cells at morula, blastocyte, or implantation-like developmental stage, suggestive of the establishment of an independent life cycle for cancer cells.

It has been reported that both parthenogenetic activation of oocyte in ovary^20^ and preimplantation embryos have tumorigenicity potential^2,3^. Consistent with this notion, nude mice injected with 100 ZP3^+^ cells isolated from p53^−/−^ HCT116 or PC3 cells developed tumors within 4 months, accompanied by the appearance of somatic tissues within tumors (Fig. 6c, Supplementary Fig. S4c). Altogether, our findings indicate that p53 deficiency could endow tumor cells with imitated embryonic development through activation of abnormal oogenesis and then parthenogenesis, which may significantly enhance the tumor’s ability to establish independent life cycle and immortality (Fig. 6h).

As both cancer cells and oocytes display strong independence and survival ability^1^, we then determined whether such oocyte-like large cells found in p53-null cancer cell lines provide the survival advantage for cancer under the selective pressure of genotoxic treatments, such as chemotherapy agents or γ-irradiation. Four weeks after treatment with chemotherapy agents, such as taxol, etoposide, or cisplatin, or 16 days after treatment with high dose of γ-irradiation (15 Gy), oocyte-like large cells and their derivatives with embryo-like structures survived and were enriched in p53^−/−^ HCT116 cells but were rarely detected in p53^+/+^ HCT116 cells, whereas almost all somatic tumor cells and most of earlier germ cell-like cells were eliminated both in p53^−/−^ HCT116 and p53^+/+^ HCT116 cells (Fig. 6d, Supplementary Fig. S4b). The survival cells were about 90% of large cells (oocyte-like large cells and their embryo-like derivatives) and about 10% of smaller round-shaped cells (earlier germ cell-like cells) in p53^−/−^ HCT116 (Supplementary Fig. S4b). Almost all of the survival cells were positive for DAZL (Fig. 6e), a specific marker detected from post-migratory PGCs stage to early embryo stage^15,16^. Our findings are consistent with the previous studies that earlier germ cells/germ cancer cells in testis or primodial follicles in ovary are highly sensitive to genotoxic agents treatments, whereas sperm and larger oocytes are resistant to these treatments ^12,29,30^.

Interestingly, large cells survived and enriched in tumor tissues under genotoxic agent treatments have been observed, but the identities and potential functions of these large cells are not well understood^5,27^. We found that the survival oocyte-like large cells and their embryo-like derivatives were alive and about 50–70% of embryo-like structures could further develop into new offspring cancer cells in semisolid medium (Fig. 6f, Supplementary Fig. S4d). Consistent with this, the survival oocyte-like large cells and their embryo-like derivatives could give rise to tumors within 4 months when inoculated to nude mice (Fig. 6g), indicative of their potential in causing tumor relapse after genotoxic therapy. Compared with tumors from p53^−/−^ HCT116 cells, the tumors derived from survival large cells (100 cells/mouse) grew slowly and were frequently enriched with oocyte-like large cells and embryo body-like structures (Fig. 6g, Supplementary Fig. S4e). It was documented that the daughter cells derived from the embryo-like giant tumor cells could cause many tumor types, including germ cell tumors^31^ as well as the number of similar large tumor cells increased in clinical post-chemotherapy specimens^31^. Collectively, our data suggest that abnormal oogenesis and their embryonic derivatives could endow tumors with strong survival ability and may contribute to the therapeutic resistance and cancer recurrence upon p53 loss.

## Discussion

A series of germ cell-like cells and preimplantation embryo-like structures observed in our findings are likely a series of malignant counterparts existing in embryo/germ cell developmental axis. We showed that p53 deficiency elicits abnormal gametogenesis from early germ cell-like stage to late oocyte-like stage and subsequent parthenogenetic activation (Fig. 6h). We therefore postulated that such process may lead to abnormal asexual reproduction and endow tumors with high embryo/germ cell developmental features. This unique reproduction-like strategy may help somatic tissue-derived cells regain the germ cell-like fate in order to establish an independent life cycle, which likely increases their independence, immortality, and survival advantage to keep “seed” in diverse environmental stresses. Importantly, the resulting PGC-like cells from such process may also facilitate tumorigenesis, resembling the natural “seed” embryonic stem cells and early PGCs in wrong place^16^, induced “seed” induced pluripotent stem cells^32^ and embryonic germ cells^16^ as well as bad seed embryonic carcinoma cells^16^. Our data indicate that a series of embryo/germ cell-like cancer cells at distinct developmental stages may contribute to distinct malignant behaviors of tumors^2,3^. It is likely that cancer initiation may be achieved from PGC-like cancer cells, whereas chemotherapy and radiotherapy resistance may be caused by the emergence of late oocyte-like large cancer cells (Fig. 6h). Our study offers the strong evidence to support the provocative embryonal/gametogenesis hypothesis of tumors through in vivo xenograft models and genetic knockout approaches. Thus, the cancer subpopulations with the traits of embryo/germ cell-like developmental axis in tumors might serve as promising targets for tumor intervention.

Using genetic mouse models, we identified germ cell-like cells are highly enriched in various types of tumors and BMDCs in p53^−/−^ mice. Interestingly, we found that germ cell-like cells, but not non-germ cell-like cells, isolated from thymic lymphomas and BMDCs in p53^−/−^ mice display stronger tumorigenicity potential, suggesting that the occurrence of germ cell-like cells are likely tumor origins that contribute to tumor development in p53^−/−^ mice. The novel tumor suppressive function of p53 as a critical barrier to restrict abnormal gametogenesis identified in this study is fully different from those classic regulation functions, such as cell cycle arrest, apoptosis, senescence, and genomic stability^11^. This new property imposed by p53 loss might have an intimate link to the previous study revealing that loss of p53 facilitates the efficiency of reprogramming from somatic cells^33^. Our findings suggest that tumor is likely not only a disease of abnormal cell growth but also a disease of abnormal reproduction^2,3,34^, offering the new mechanistic insight into the p53 regulatory network in tumor suppression. As germ cell-like cells display the tumorigenicity potential and the crucial nature of life is reproduction, it is likely that activation of abnormal gametogenesis may serve as a more potent driving force to support tumor initiation than abnormal cell growth.

The *p53* gene family, which is broadly conserved over evolutionary timescales, mediates adaptive responses to genotoxic stress^35,36^. It is thought that tumor suppression may not be primary function for the p53 family genes, since homologs of the p53 family genes appear in lower organisms, such as flies and worms, which do not develop tumors partly due to their short life span^35,36^. It is proposed that maintaining the integrity of the germline and reproduction may be the primary functions for the ancestral p53 family genes^35,36^. Supporting evidence came from the earlier reports showing that deletion of the *p53*, *p63*, or *p73* gene in female mice causes a significant decrease in fertility^28,35,36^. However, compared with p63 and p73, p53 plays a less important role in maintaining the integrity of the genome in germ cells, but more important roles in tumor suppression^35,36^. The key role of p53 in restricting abnormal gametogenesis identified in our study further underscores the role of *p53* gene in protecting normal gametogenesis and reproduction for tumor suppression. This unique function of p53 as a guardian in normal gametogenesis and reproduction not only contributes to the possible ancestral role of p53 family genes but also offers a plausible explanation for the evolution of the *p53* gene to obtain dual roles in tumor suppression and reproduction maintenance, thus filling the evolutional gap of *p53* gene family.

The embryonal/germ cell traits of tumors are likely the most common traits of tumors^1–3^. The expression of embryonal/germ cell-related markers is extensively detected in a variety of cancer types, and some of these markers, such as AFP and HCG are used as biomarkers for the clinical diagnosis of tumors^2,3^. Interestingly, the embryo/germ cell-related markers, such as Oct4, Sox2, Nanog, and SSEA1^23–26^, are often used as markers for cancer stem cells (CSCs), which are believed to play a vital role in tumor initiation, progression, and metastasis. We postulate that PGC-like cells are similar to CSCs in certain aspects, but they do display some distinct properties. For the “stemness” of tumors, the concept of CSCs suggests that a subpopulation of tumor cells display tumorigenicity potential, self-renewal capability, and the ability to differentiate into somatic tumor cells, similar to PGC-like cells. However, CSCs may not have the similar properties such as the generation of embryonic germ cell-like tumor cells (multipotent), oocyte-like cells, and the formation of preimplantation embryo-like structures presented in PGC-like cells. In particular, PGC-like tumor cells upon p53 deficiency could establish the independent life cycle through their germ cell-like development, indicative of a real “stemness”. Polyploid giant cancer cells include oocyte-like cancer cells and preimplantation embryo-like cancer cells. Abnormal gametogenesis and parthenogenetic activation in tumors endow the giant cancer cells with a new cell fate. The giant cancer cells frequently observed in high grade of human cancers and enriched after the treatment of chemotherapeutical drugs and γ-irradiation^31^ display not only a pathologic morphology as a passenger but also constitute biological functions driving cancer with a new way. Therefore, both CSCs and polyploid giant cancer cells possibly represent a short stage of the embryonal/germ cell-like developmental axis, respectively. In contrast, the embryonal/gametogenesis found in tumors presented in this study could link these distinct features of cancer together.

Our findings demonstrate that distinct malignant traits of tumors attribute to the occurrence of tumor cells at the different embryo/germ cell-like developmental stages. We rationalize that targeting the entire embryonal/germ cell-like developmental axis for tumor intervention is likely a better strategy than targeting CSCs, especially for those tumors at late stage with p53 functional defects.

## Methods

Key resources table

**Table:** 

Reagent or resource	Source	Identifier
Antibody		
4-Oct	Abcam	Cat no: ab184665
Sox2	R&D	Cat no: MAB2018R-100
Vasa	Abcam	Cat no: ab27591
DAZL	Novus Biologicals	Cat no: NB100-2437
Nanos3	Abcam	Cat no: ab70001
GDF9	R&D	Cat no: AF739
SCP3	Abcam	Cat no: ab15093
Nanog	Novus Biologicals	Cat no: NB100-58842
Stellar	Fisher	Cat no: PA5-34601
Prdm14	Abcam	Cat no: ab187881
Ifitm3-Alexa 488	Abcam	Cat no: ab198559
Ifitm3	Abcam	Cat no: ab109429
ZP3	Proteintech Group Inc.	Cat no: 21279-1-AP
p53	Santa Cruz	Cat no: sc-126
Genotyping mice		
Oct4-GFP knock-in reporter mice	Jackson laboratory	S4-*Pou5f1*^*tm2Jae*^/J
p53^+/−^ mice	Provided by Dr. E. Flores at MD Anderson Cancer Center	
Plasmid		
p53-shRNA	Sigma	Cat no: TRCN0000003753
		Cat no: TRCN0000342259
pLenti6/V5-p53	Invitrogen	Cat no: 22936

### Animal models

p53^+/−^ mice in C57BL/6 background, kindly provided by Dr. E. Flores at MD Anderson Cancer Center, were crossed with Oct4-GFP knock-in reporter mice, which harbor IRES-GFP fusion cassette downstream of the stop codon of the Oct4 (S4-*Pou5f1*^*tm2Jae*^/J) gene (Jackson laboratory, Cat: 008214)^37^, and the resulting mice were further intercrossed to generate p53^−/−^ Oct4-GFP^+/+^ and p53^+/+^ Oct4-GFP^+/+^ mice (Supplementary Table S3) for primary cell cultures. The p53^−/−^ mice and p53^+/+^ mice with mixed inbred C57BV6 × 129S4 background were used to observe the abnormal gametogenesis and the spontaneous tumor formation. All mice were monitored for tumor phenotypes three times a week up to the age of 12 months before all of the surviving animals were sacrificed. Moribund animals or those mice developing obvious tumors before this end point were also sacrificed and necropsied. The p53^−/−^ mice were sacrificed and subjected to necropsy when they showed the signs of possible thymic lymphoma development, as described previously^21,22^. The tumors were placed in 10% neutral buffered formalin (NBF) for further histopathological analysis. All animal procedures and studies were conducted in accordance with the Institutional Animal Care and Use Committee from MD Anderson Cancer Center and Wake Forest School of Medicine.

### Genotyping

Genomic DNA from tail biopsies was genotyped by PCR using the primers listed in the Extended-table3.

### Cell culture

BMDCs were isolated from the (femur and tibia) bones of 4-week-old Oct4-GFP^+/+^ p53^−/−^ or Oct4-GFP^+/+^ p53^+/+^ mice and cultured in low-glucose Dulbecco’s modified Eagle’s medium (DMEM, Hyclone) with 10% fetal bovine serum (FBS; Sigma) and 1% l-glutamine. One week after culture, nonadherent cells were discarded and surviving adherent cells were kept and referred to as BMDCs, which were observed and taken pictures under microscope (Nikon). RKO, PC3, U2OS, HepG2, Hep3B, LNCaP, and H1299 were purchased from American Type Culture Collection. p53^+/+^ HCT116 and p53^−/−^ HCT116 cells were obtained from Dr. M.H. Lee at MD Anderson Cancer Center. All human cancer cell lines were cultured in DMEM high glucose (Hyclone) containing 10% FBS. Cultured cells were observed and collected at different days to study the formation of PGC-like cells and gene expression. Germ cell-like cells appeared spontaneously in the induced p53^−/−^ BMDCs and then underwent proliferation, migration, and further differentiation into larger germ cell-like cells and embryo-like structure formation in regular medium under microscope. Stellar^+^ cells sorted from thymic lymphomas or total cells isolated from thymic lymphoma from p53^−/−^ mice were cultured in DMEM high glucose (Hyclone) containing 10% FBS. In order to observe the formation of embryo-like structures from germ cell-like cells, approximately 1000 suspending cells were plated in a semisolid medium. All cells were maintained at 37 °C with 5% CO2. The medium was changed twice a week. In routine culture, the cells would be subcultured when they reached 90–100% confluence.

### Semisolid culture

In order to better observe the formation of embryo-like structures form germ cell-like cells, various human cancer cell lines were plated in a semisolid medium containing 2.7% methyl cellulose (Sigma), high-glucose DMEM (Invitrogen), and 10% FBS (Sigma), half of which was replenished with fresh medium every week for up to 30 days.

### Real-time PCR analysis

RNA was isolated from various cell types using Trizol reagent (Invitrogen) and then subjected to reverse transcription with a reverse transcription kit (Invitrogen), according to the manufacturer’s protocol. Real-time PCR analysis was performed by using the SYBR Green PCR Master Mix Kit (Applied Biosystems). Primers and expected product size were listed in the Supplementary Table S4.

### Xenograft tumor formation and analysis

The sorted Oct4-GFP^+^ (100 cells) or Oct4-GFP^−^ (100 cells) from p53^−/−^ BMDCs or p53^+/+^ BMDCs (5 × 10^5^ cells) with six passage culture were injected subcutaneously into nude mice (five mice/group). The mice injected with Oct4-GFP^+^ p53^−/−^ BMDCs were sacrificed when the diameter of tumors was close to 1.5 cm, while those injected with Oct4-GFP^−^ p53^−/−^ BMDCs and p53^+/+^ BMDCs were not sacrificed until 6 months after injection. To identify tumor subpopulation similar to PGCs in function, p53^+/+^ HCT116, p53^−/−^ HCT116, RKO, PC3, Hep3B, or H1299 cells (1 × 10^5^ cells) were injected subcutaneously into nude mice (five mice/group) and the mice were sacrificed when the tumor reached to 1.5 cm in diameter. To define the tumorigenicity potential of germ cell-like cells isolated from human cancer cell lines, the sorted Ifitm3^+^ cells (100 cells) from p53^+/+^ HCT116, p53^−/−^ HCT116, RKO, and PC3 cells or ZP3^+^ cells (100 cells) from p53^−/−^ HCT116 and PC3 cells were injected subcutaneously into nude mice (five mice/group), and the mice were sacrificed when the diameter of tumors was close to 1.5 cm. In all, 1 × 10^5^ cells from two different thymic lymphomas were injected subcutaneously into nude mice (*n* = 3), and the mice were sacrificed 4 weeks after injection. The sorted Stellar^+^ (100 cells) or Stellar^−^ (100 cells) cells from thymic lymphomas of p53^−/−^ mice were injected subcutaneously into nude mice (three mice/group) for monitoring tumor development. Two independent lymphomas were sorted and injected subcutaneously into nude mice, and the mice were sacrificed 6 weeks after injection. Stellar^+^ thymic lymphoma (1 × 10^5^ cells) after 30-day culture sorted from p53^−/−^ mice were injected subcutaneously into nude mice (*n* = 3), and the mice were sacrificed when tumor reached 1 cm in diameter or 12 months after injection. Total cells (1 × 10^5^ cells) after 21-day culture isolated from thymic lymphoma from p53^−/−^ mice were injected subcutaneously into nude mice (*n* = 3), and the mice were sacrificed when tumor reached 1 cm in diameter or 12 months after injection. For tumorigenicity analysis of surviving cells, 100 or 1000 surviving p53^−/−^ HCT116 cells 4 weeks after taxol treatment were injected subcutaneously into nude mice (five mice/group) for monitoring tumor development. The caliper was used to measure the tumor size, and the tumor volume was calculated by the equation: [mm^3^] = (length [mm]) × (width [mm])^2^ × 1/2.

### Histology and immunohistochemistry

Tumor tissues were fixed with 10% NBF overnight, embedded in paraffin, and sectioned according to standard tissue-processing protocol. Formalin-fixed paraffin-embedded tissue sections were stained with hematoxylin and eosin or used for immunohistochemical staining. For immunohistochemistry, the section slides were antigen retrieved (0.01 M Citrate buffer, pH 6.0), stained with indicated primary antibody for 2 h, and then detected by VECTASTAIN Universal Quick HRP Kit (Peroxidase) (Vector laboratory) with DAB kits (Abcam). Hematoxylin staining was used to show nuclear details. We stained Oct4 expression in lymphomas from 40 mice, teratomas in testis tumors from 11 mice, sarcomas from 14 mice, and normal thymi from 6 mice. We counted 1000 lymphomas cells under microscope to obtain the ratio of Oct4^+^ in lymphomas after Oct4 staining. Six lymphomas were used to analyze the ratio of Oct4^+^ cells.

### Immunofluorescence

For immunofluorescence, cultured cells were washed twice with phosphate-buffered saline (PBS), subsequently fixed with 4% paraformaldehyde, permeabilized for 10 min with 0.3% Triton-X-100, blocked in PBS with 2.5% bovine serum albumin and 0.05% Triton-X-100, and then stained with primary antibodies overnight at 4 °C. Next, the cells were washed, stained with fluorescent dye-conjugated secondary antibodies (Alexa Fluor 488, Alexa Fluor 555; Invitrogen), 4, 6-diamidino-2-phenylindole (Invitrogen), and taken pictures with microscope.

### AP staining

Cultured cells were fixed with 4% paraformaldehyde for 4 min, washed twice with a Tris-HCl (pH = 8.2) buffer solution, and then incubated with AP staining solution (Vector Laboratories) according to the manufacturer’s protocol.

### Immunoblotting

For immunoblotting, cells were lysed in RIPA buffer containing proteinase inhibitor cocktail and phosphatase inhibitors (10 mM Na-pyrophosphate, 10 mM Naglycerophosphate, and 50 mM Na-fluoride) and subjected to immunoblotting by indicated antibodies.

### Fluorescence-activated cell sorting and flow cytometric analysis

To isolate Stellar^+^ or Stellar^−^ cells, the thymic tumor cells were isolated by mechanical disruption of thymic tumor obtained from p53^−/−^ mice using two cover slides, filtered, and treated with blood cell lysed buffer (Sigma). The cells were then incubated with Stellar antibodies for 1 h on ice and incubated with Alexa Fluor 488-conjugated secondary antibodies (Invitrogen), and subjected to fluorescence-activated cell sorting (FACS). To isolate Oct4-GFP^+/+^ and Oct4-GFP^−/−^ BMDC cells, BMDCs were subjected to FACS by GFP fluorescence signal. To isolate Ifitim3^+^ or Ifitim3^−^ cells, the cultured cancer cells were incubated with Ifitm3-Alexa Fluor 488 antibody for 1 h on ice, subjected to FACS. To isolate ZP3^+^ cells, the cultured cancer cells were incubated with ZP3 primary antibody for 1 h on ice, followed by incubating with Alexa Fluor 488-conjugated secondary antibody and subjected to FACS. Antibodies against mouse immunoglobulin conjugated to Alexa Fluor 488 were used as antibody isotype controls (BD). The labeled cells were analyzed on the FACS Vantage SE flow cytometer (BD).

### Lentiviral infection

For lentiviral infection, 293T cells were co-transfected with lentiviral plasmids (pLKO-puro or pLenti6/V5), packing plasmid (deltaVPR8.9), and envelope plasmid (VSV-G). Retrovirus-containing medium was harvested at 48 and 72 h after transfection, filtered through a 0.45 μm filter, and used to infect cancer cell lines. All the infected cells were cultured in the medium with 2 μg/ml puromycin (Sigma) or 4 μg/ml blasticidin (Invitrogen) for 1 week before further analysis. Short interfering RNA for p53 (Supplementary Table S5) and luciferase were purchased from Sigma. pLenti6/V5-p53 was purchased from Invitrogen. After transfection with p53 expressing vector and selection for 1 week in high-glucose DMEM with 10% FBS and 4 μg/ml blasticidin, multiple single-cell clones of p53^−/−^ HCT116 cells were isolated and cultured in 96-well plates. Confluent cells were then plated in 6-well plates for immunoblotting and oocyte-like large cell determination.

### Genotoxic treatment

p53^+/+^ HCT116 and p53^−/−^ HCT116 cells were cultured in the six-well plates (5 × 10^5^ cells/well) and treated with taxol (50 nM, Sigma), etoposide (40 μM, Sigma), or cisplatin (40 μM, Sigma). Four weeks after treament, the ratio of the surviving cells was counted, and these surviving cells were cultured in the semisolid medium or injected into nude mice. p53^+/+^ HCT116 and p53^−/−^ HCT116 cells were cultured in the six-well plate (5 × 10^5^cells/well) and treated with 15 Gy γ-irradiation. After treatment with γ-irradiation, the p53^+/+^ HCT116 and p53^−/−^ HCT116 cells were further cultured for 16 days and then the ratio of the surviving cells was counted. These surviving cells were also cultured in the semisolid medium to examine the ratio of formation of clones with somatic cells.

### Cell counts

To determine the percetages of oocyte-like large cells in cultured cancer cell lines, various cancer cell lines were cultured in six-well plates for 5 days, and the number of oocyte-like large cells and total cancer cells were counted under the microscope and a cell counter machine, respectively. The surviving cancer cells after treatment with chemotherapeutic agents or γ-irradiation in six-well plates were counted under the microscope.

### Statistical analysis

All data are shown as means ± s.d. from at least three independent experiments. All statistical significance was determined by unpaired two-tailed Student’s *t*-tests. Results with *p*-value < 0.05 for all tests were considered significant.

## Electronic supplementary material

Supplementary Information

Supplementary Movie S1

Supplementary Movie S2

Supplementary Movie S3

## Electronic supplementary material

**Supplementary Information** accompanies the paper at (10.1038/s41421-018-0054-x).
