# Supplementary material for: Abnormal gametogenesis induced by p53 deficiency promotes tumor progression and drug resistance
Source: Cell Discov. 2018 Oct 2;4:54. doi: 10.1038/s41421-018-0054-x (PMC6167385; doi:10.1038/s41421-018-0054-x)
Supplement: Supplementary file 1 — Supplementary Information [file 41421_2018_54_MOESM1_ESM.pdf]

Supplemental Information

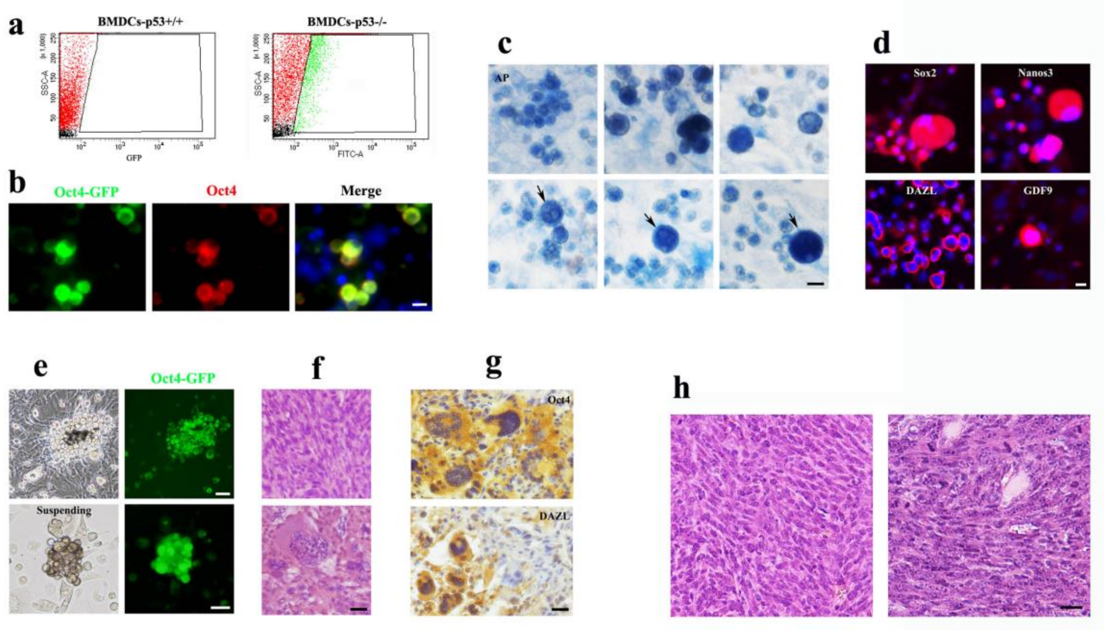

Supplementary Figure S1. Traits of germ cell-like cells are present in $p53^{-/-}$ BMDCs. a, Representative FCS of paired $p53^{+/+}$ (<0.1%) and $p53^{-/-}$ (33.76±0.72 %, n=3) primary BMDCs culture 4 weeks after the culture were shown. **b,** Oct4-GFP fluorescence, Oct4 immunofluorescence staining and DAPI staining image in $p53^{-/-}$ BMDCs. **c**, Cultured $p53^{-/-}$ BMDCs were stained with AP. The germ cell-like cells at different developmental stages and early embryo-like structures (arrow) were positive for AP staining. **d**, $p53^{-/-}$ BMDCs were stained with antibodies for distinct germ cell specific markers. Germ cell-like cells at different developmental stage were positive for germ cell-related markers. **e**, Bright field and Oct4-GFP immunofluorescence image of cell sphere attached to the plate or suspending in medium in $p53^{-/-}$ BMDCs cultures. **f**, Representative tumour sections derived from Oct4-GFP^{+} $p53^{-/-}$ BMDCs with H&E staining showed sarcoma characteristics. **g**, Representative tumour sections from Oct4-GFP^{+} $p53^{-/-}$ BMDCs stained with the antibody against Oct4 or DAZL were shown. Oocyte-like cells in sarcomas were positive for Oct4 and DAZL. **h**, Representative tumour sections derived from the other two Oct4-GFP^{+} $p53^{-/-}$ BMDCs with H&E staining showed sarcoma characteristics. (Scale bar=20 μm in b, c, f, g, h, 10 μm in d)

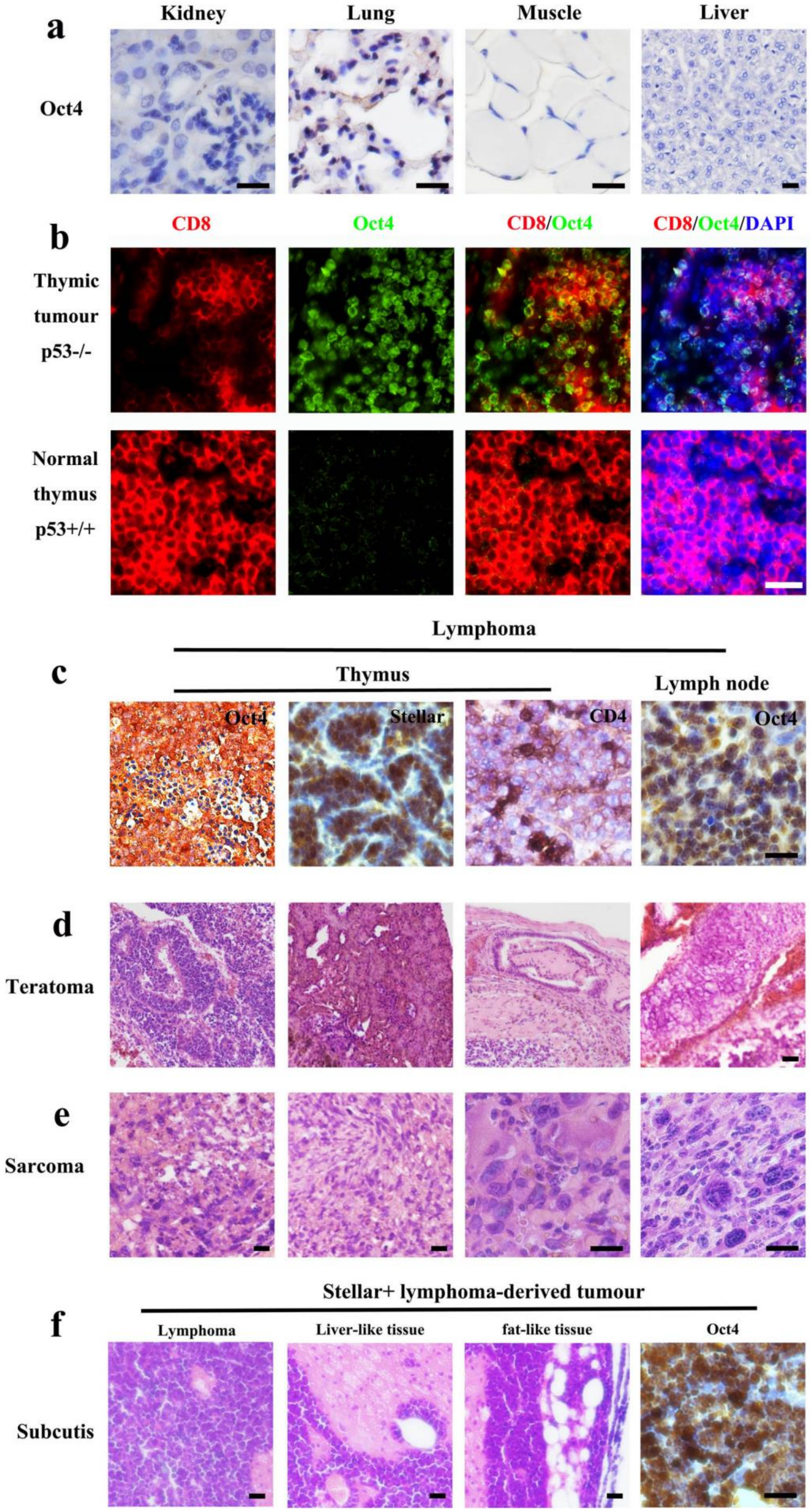

a
Kidney
Lung
Muscle
Liver
Oct4
b
CD8
Oct4
CD8/Oct4
CD8/Oct4/DAPI
Thymic tumour p53-/-
Normal thymus p53+/+
Lymphoma
c
Thymus
Lymph node
Oct4
Stellar
CD4
Oct4
d
Teratoma
e
Sarcoma
Stellar+ lymphoma-derived tumour
f
Lymphoma
Liver-like tissue
fat-like tissue
Oct4
Subcutis

20

Supplementary Figure S2. Germ cell-like cells are enriched in distinct tumours from p53$^{-/-}$ mice and xenograft tumours. **a**, Sections of normal tissues from p53$^{-/-}$ mice without obvious tumours were stained with Oct4 antibody. **b**, Sections of paired thymic lymphoma of p53$^{-/-}$ mouse and normal thymus of p53$^{+/+}$ mouse were stained with H&E or antibodies against the indicated proteins. **c**, Sections of thymic lymphoma from p53$^{-/-}$ mice were stained with antibodies against the indicated proteins. **d**, Sections of spontaneous teratomas from p53$^{-/-}$ mice were stained with H&E. **e**, Sections of spontaneous sarcomas from p53$^{-/-}$ mice were stained with H&E. The sarcomas showed different subtypes. **f**, The tumour section derived from sorted Stellar^{+} tumour cells of thymic lymphoma was stained with H&E or Oct4 antibody. (Scale bar=20 μm)

a

Ratio of oocyte-like cells in different cancer cell lines			
**Cell line**	**p53 genotype**	**Ratio of oocyte-like large cells (1/1000000)**	**Ratio of ZP3$^+$ cells (%)**
RKO	p53$^{+/+}$	3±1	0.07±0.06
LNCAP	p53$^{+/+}$	12.33±1.52	1.30±0.10
U2OS	p53$^{+/+}$	0±0	0.13±0.06
HepG2	p53$^{+/+}$	0±0	0.23±0.06
Hep3B	p53$^{-/-}$	165.67±38.94	1.67±0.21
PC3	p53$^{-/-}$	1290±121.86	3.00±0.20
H1299	p53$^{-/-}$	208.33±28.29	0.47±0.06

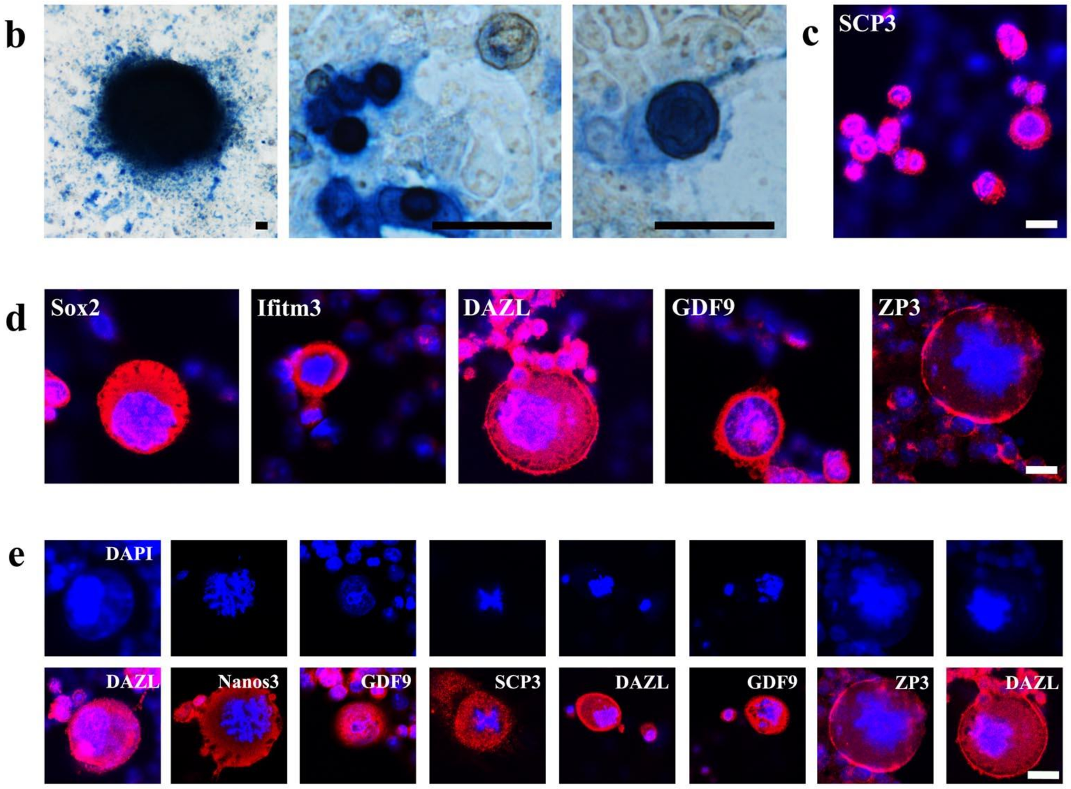

f

Ability of oocyte-like large cell formation in HCT116p53-/- over expressing p53wt (1/1000000)							
**Clone**	**B**	**C**	**D**	**G**	**I**	**J**	**K**
No.	10±1.73	24.3±2.5	12±2	142.3±7.5	8.7±2.1	65±7	269.3±16.3

Supplementary Figure S3. p53 deficiency promotes oocyte-like large cell formation. a, The relative ratio of oocyte-like large cells and ZP3$^+$ cells in diverse cancer cells were determined (n=3). **b**, The p53$^{-/-}$ HCT116 cells were stained with AP. Germ cell-like cell clusters and germ cell-like cells at the developmental stage could be observed during cultures. Germ cell-like cell cluster and early germ cell-like cells were positive for AP staining while the late germ cells were negative (arrow). **c**,

Immunostaining showed the expression of SCP3, a meiotic marker in HCT116p53$^{-/-}$ cultures. **d**, Oocyte-like cells were stained with antibodies against the indicated proteins. **e.** The oocyte-like cells stained with DAPI and antibodies against the indicated proteins resembling oocyte at different meiotic stage. **f**, The relative ratio of oocyte-like large cells in p53$^{-/-}$ HCT116 cells with or without p53 restoration from multiple single-cell clones were analyzed (n=3). (Scale bar=50μm in b, 20 μm in d, 25 μm in e)

a

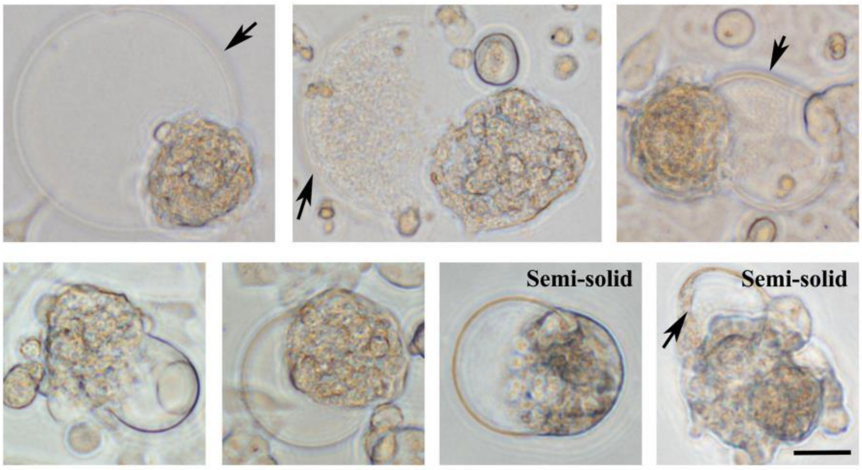

b

Difference of survival ability					
Tumour cell	Survival tumour cells	Taxol	Etapsite	Cisplatin	Irradiation
HCT116P53-/-	Oocyte-like large cells	476.3±26.1**	401±11.5**	774.6±32.1**	1501.7±56.7**
HCT116P53+/+		0.7±0.6	0.3±0.6	0.3±0.6	0.3±0.6
HCT116P53-/-	Smaller tumour cells	59±23.6	19±3.6	122.6±9.3**	145±17.9**
HCT116P53+/+		29.3±5.5	19.3±3.2	53.3±11.9	12.6±2.1
HCT116P53-/-	Total tumour cells	535.3±49.9**	420.7±13.6**	830.6±27.2**	1647±39.1**
HCT116P53+/+		30±5.6	19.7±3.1	53.7±11.4	13±2

(** p<0.01)

c

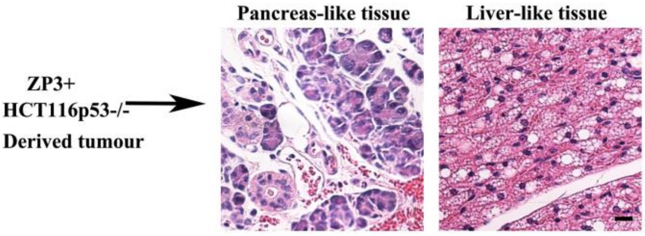

d

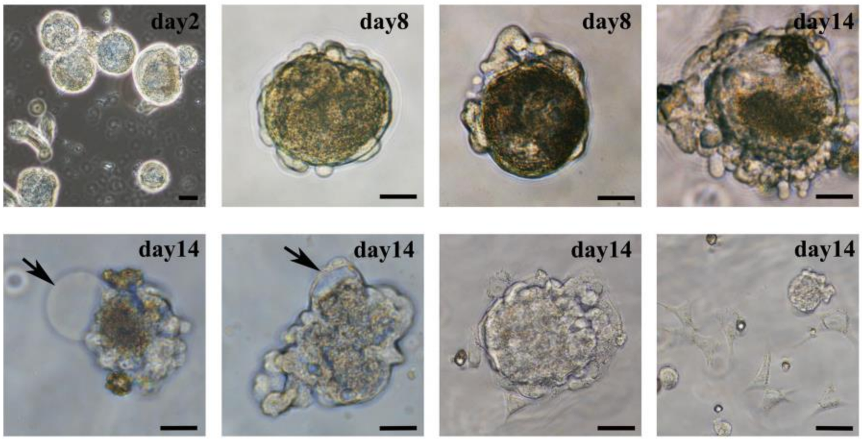

e

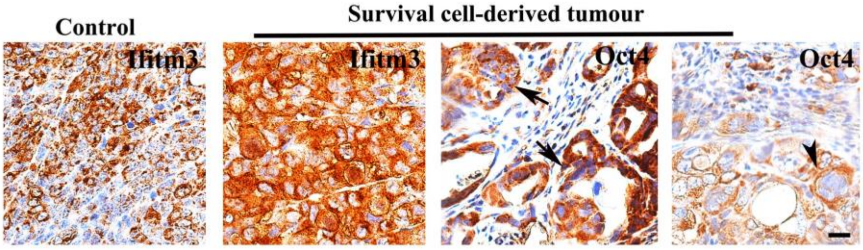

Supplementary Figure S4. Abnormal oogenesis contributes to cancer cell resistance to diverse genotoxic stresses. a, Bright field images of implatation or post-implatation-like embryonic derivatives from HCT116 $p53^{-/-}$ cells cultured in regular medium or semi-soild medium. The new offspring cells were observed to derive from embyo-like structures. The arrow indicated the zona pellucida-like membrane. **b**, The relative number of surviving cells from paired $p53^{+/+}$ and $p53^{-/-}$ HCT116 cells 4 weeks after distinct genotoxic treatment or 16 days after γ-irradiation was shown (n=3). **c**, Tumour sections from nude mice subcutaneously injected with the $ZP3^{+}$ cells sorted from $p53^{-/-}$HCT116 cells were stained with H&E. **d**, Oocyte-like large cells and their derivatives survived after Taxol treatment were cultured in semi-solid medium. Bright field images of the spheres at different time points of culture were shown. Some spheres could attach to the plate and give rise to the somatic tumour cells. **e**, Tumour sections from nude mice with subcutaneous injection of 100 surviving large $p53^{-/-}$ HCT116 cells 4 weeks after treatment with taxol or untreated $p53^{-/-}$ HCT116 cells (control) were stained with antibodies against the indicated proteins. Bigger germ cell-like cells, embryonic body-like structures (arrow) and oocyte-like large cells (arrowhead) were readily observed in the tumours from the surviving cells. (Scale bar= 20 μm).

Supplementary Table S1. The table shows the relative ability of germ cell formation in primary $p53^{+/+}$ BMDCs and $p53^{-/-}$ BMDCs cultures after cultured 4 weeks (n=3).

**Difference of germ cell-like cell formation between $p53^{-/-}$ and $p53^{+/+}$ mice**						
**Mixed C57BL/6 × 129/Sv genetic background**	**Age (week)**	**Gender**	**Clusters in cultures (>30 cells) n=3**	**Ratio of Oct4-GFP+ (%) n=3**	**Ratio of oocyte-like large cells (1/1,000,000) n=3**	**Tumourigenicity**
$p53^{-/-}$Oct4-$GFP^{+/+}$-1#	4	Male	217±8.0	3.83±0.25	124±6.5	Yes
$p53^{-/-}$Oct4-$GFP^{+/+}$-2#	4	Female	98±12.3	1.33±0.68	43±6.5	Yes
$p53^{-/-}$Oct4-$GFP^{+/+}$-3#	4	Female	502±23.3	33.76±0.72	704±124.9	Yes
$p53^{+/+}$Oct4-$GFP^{+/+}$-1#	4	Male	1	<0.1	1	No
$p53^{+/+}$Oct4-$GFP^{+/+}$-2#	4	Female	0	<0.1	1	No

$p53^{+/+}$Oct4-GFP$^{+/+}$-3#	4	Female	0	<0.1	0	No
p			<0.01		<0.05	

Supplementary Table S2. Tumour Spectrum in C57BL/6 × 129/Sv $p53^{-/-}$ mice.

	Age	Gender	Location	Tumour type	Large spleen	Oocyte-like cells	Mature tissue
1	15	F	Thymus	Lymphoma	No	No	No
2	15	M	Thymus	Lymphoma	No	No	No
3	16	M	Thymus	Lymphoma	No	No	Yes
4	24	F	Thymus	Lymphoma	No	No	Yes
5	10	M	Thymus	Lymphoma	No	No	No
6	8	F	Thymus	Lymphoma	No	No	No
7	28	M	Subcutis-lymph node	Lymphoma	Yes	No	Yes
8	16	M	Thymus	Lymphoma	No	No	No
9	14	M	Thymus	Lymphoma	No	No	No
10	21	M	Thymus	Lymphoma	No	No	No
11	18	F	Thymus	Lymphoma	No	No	Yes
12	21	F	Thymus	Lymphoma	No	No	No
13	23	M	Thymus	Lymphoma	Yes	No	Yes
14	19	M	Thymus	Lymphoma	No	No	No
15	20	M	Subcutis-lymph node	Lymphoma	No	No	No
16	26	M	Thymus	Lymphoma	No	No	Yes
17	20	M	Thymus	Lymphoma	No	No	No
18	15	M	Thymus	Lymphoma	No	No	Yes
19	17	F	Thymus	Lymphoma	No	No	No
20	13	F	Thymus	Lymphoma	No	No	Yes
21	21	M	Thymus	Lymphoma	No	No	No
22	23	M	Thymus	Lymphoma	No	No	No
23	19	M	Thymus	Lymphoma	Yes	No	Yes
24	28	F	Subcutis-lymph node	Lymphoma	No	No	No
25	15	F	Thymus	Lymphoma	No	No	Yes
26	15	F	Thymus	Lymphoma	No	No	No
27	15	M	Thymus	Lymphoma	No	No	No
28	16	M	Thymus	Lymphoma	No	No	No
29	21	M	Thymus	Lymphoma	No	No	Yes
30	18	M	Thymus	Lymphoma	No	No	No
31	20	M	Thymus	Lymphoma	No	No	No
32	17	M	Thymus	Lymphoma	No	No	Yes

33	9	M	Thymus	Lymphoma	No	No	No
34	21	F	Thymus	Lymphoma	No	No	No
35	20	F	Thymus	Lymphoma	No	No	No
36	16	M	Thymus	Lymphoma	No	No	No
37	7	M	Testis	Malignant teratoma	No	No	Yes
38	5	M	Testis	Malignant teratoma	No	No	Yes
39	4	M	Testis	Malignant teratoma	No	No	Yes
40	3	M	Testis	Malignant teratoma	No	No	Yes
41	4	M	Testis	Malignant teratoma	No	No	Yes
42	8	M	Testis	Malignant teratoma	No	No	Yes
43	7	M	Testis	Malignant teratoma	No	No	Yes
44	17	M	Mouth	Sarcoma	Yes	No	No
45	9	M	Subcutis	Sarcoma	No	No	Yes
46	16	F	Leg	Sarcoma	Yes	No	No
47	24	M	Subcutis	Sarcoma	No	No	Yes
			Bone	Sarcoma		No	No
48	14	M	Leg	Sarcoma	No	No	No
49	13	M	Subcutis	Sarcoma	Yes	No	No
50	20	M	Subcutis	Sarcoma	Yes	No	Yes
51	18	M	Subcutis	Sarcoma	No	Yes	Yes
52	17	F	Abdomen	Malignant teratoma	No	Yes	Yes
53	13	M	Abdomen	Malignant teratoma	Yes	No	Yes
54	20	F	Abdomen	Malignant teratoma	Yes	No	Yes
55	13	M	Abdomen	Malignant teratoma	No	No	Yes
56	20	M	Left leg	Sarcoma	No	No	Yes
			Thymus	Lymphoma	No	No	Yes
			Abdomen	Malignant teratoma	No	No	Yes
57	6	M	Subcutis	Sarcoma	Yes	No	No
			abdomen	Malignant teratoma	No	No	Yes
58	16	F	Subcutis	Sarcoma	Yes	No	No
			Thymus	Lymphoma	No	No	No

59	16	M	Left leg	Sarcoma	No	No	Yes
			Thymus	Lymphoma	No	No	No
60	19	F	subcutis	Sarcoma	No	No	No
			Thymus	Lymphoma	No	No	No

Supplementary Table S3. Primer of genotyping detection.

**Primer**	**Sequence 5'-3'**	**Size**
Oct4-Common	CAA GGC AAG GGA GGT AGA CA	Mutant = 234 bp<br>Heterozygote = 434 bp and 234 bp<br>Wild type = 434 bp
Oct4-Wild type	TGC CAG ACA ATG GCT ATG AG	
Oct4-mutant	CCA AAA GAC GGC AAT ATG GT	
p53-X7	TATACTCAGAGCCGGCCT	Mutant = 650 bp<br>Heterozygote = 650bp and 450 bp<br>Wild type = 450bp
p53-neo18	CTATCAGGACATAGCGTTGG	
p53-X6.5	ACAGCGTGGTGGTACCTTAT	

Supplementary Table S4. Primer for QRT-PCR.

Gene	Forward/reverse	Sequence 5'-3'	Base pairs
hqOct4	forward	GCTGGAGCAAAACCCGGAGG	114
	reverse	TCGGCCTGTGTATATCCCAGGGTG	
hqNanog	forward	TGAACCTCAGCTACAAACAG	154
	reverse	TGGTGGTAGGAAGAGTAAAG	
hqStellar	forward	ACGCCGATGGACCCATCACAGTTT	112
	reverse	TCTCGGAGGAGATTTGAGAGGCCC	
hqGDF3	forward	AAATGTTTGTGTTGCGGTCA	179
	reverse	TCTGGCACAGGTGTCTTCAG	
hqTNAP	forward	AAGCAGGTCTTGGGGTGCACCA	117
	reverse	TTGGTCTCGCCAGTACTTGGGGT	
hqPrdm1	forward	CGGGGAGAATGTGGACTGGGTAGAG	110
	reverse	CTGGAGTTACACTTGGGGGCA	

		GC	
hqSCP3	forward	CTAGAATTGTTCAGAGCCAGAGA	247
	reverse	GTTCAAGTTCTTTCTTCAAAG	
hqZP2	forward	GCCTCCCAGGACCCATTCTC	
	reverse	CAGGTAGTAGATGAAGCCTA	245
hqGADPH	forward	CAAAGTTGTCATGGATGACC	195
	reverse	CCATGGAGAAGGCTGGGG	
mqOct4	forward	TCAGGTTGGACTGGGCCTAGT	100
	reverse	GGAGGTTCCCTCTGAGTTGCTT	
mqSox2	forward	GAGGGCTGGACTGCGAACT	72
	reverse	TTTGCACCCCTCCCAATTC	
mqNanog	forward	GAAATCCCTTCCCTCGCCATC	160
	reverse	CTCAGTAGCAGACCCTTGTAAGC	
mqStellar	forward	GCAGTCTACGGAACCGCATT	123
	reverse	GGTCTTTCAGCACCGACAACA	
mqNanos3	forward	TAAGGCTGGATCCCAAACCA	115
	reverse	GACTCGCCATTGTGTTTGCA	
mqIfitm3	forward	AACATGCCCAGAGAGGTGTC	174
	reverse	CTTAGCAGTGGAGGCGTAGG	
mqSCP1	forward	AAGTTTGATTCTAAAACAACTCCTTCA	168
	reverse	ACTCTTTTTAGTTGGTGTCTTCACTGT	
mqSCP3	forward	AGAAATGTATACCAAAGCTTCTTTCAA	180
	reverse	TTAGATAGTTTTTCTCCTTGTTCCTCA	
mqDmc1	forward	GGGATACAAATGACAACAAG	239
	reverse	CGAAATTCTCCAAAAGCTTC	
mqOog1	forward	ACCTCAGGTGCCTGAAAAAG	251
	reverse	CAGCAAGGCACTGAACTGAA	
mqNobox	forward	GGACAAGGCCTATGTGTCCT	364
	reverse	GGGTCCTGTACCCATGTTTT	
mq*GAPDH*	forward	AAGGGCTCATGACCACAGTC	207
	reverse	ACACATTGGGGGTAGGAACA	

Supplementary Table S5. Sequence of p53shRNA.

TRC-number	Sequence
TRCN00000	CCGGGTCCAGATGAAGCTCCCAGAACTCGAGTTCTGGGA

03755	GCTTCATCTGGACTTTTT
TRCN0000003753	CCGGCGGCGCACAGAGGAAGAGAATCTCGAGATTCTCTTCCTCTGTGCGCCGTTTTT

Supplementary Movie S1. Time lapse of a large cell development from p53$^{-/-}$ HCT116 cells in regular medium. A round-shape large cell spread on the plate and gave rise to a multinuclear tumour giant cells, which then could release some offspring cells. Some round-shape large cells and multinuclear tumour giant cells could switch mutually. The multinuclear tumour giant cells displayed the features similar to early embryo-like cells.

Supplementary Movie S2. Time lapse of oocyte-like large cell development from p53$^{-/-}$ BMDCs cultured in semisolid medium. The time lapse showed two oocyte-like large cells gave rise to embryo-like structures and then cell spheres in the culture. Through this way, the single oocyte-like cell could give rise to new offspring cells.

Supplementary Movie S3. Time lapse of a large cell development from p53$^{-/-}$ HCT116 cells in semisolid medium. A large cell has an ability to divide and then gave rise to a cell sphere.
